# Impact of the Sars-Cov-2 outbreak on the initial clinical presentation of new solid cancer diagnoses: a systematic review and meta-analysis

**DOI:** 10.1186/s12885-023-11795-1

**Published:** 2024-01-29

**Authors:** Simon Marty, Guillaume Lamé, Etienne Guével, Sonia Priou, Gilles Chatellier, Christophe Tournigand, Emmanuelle Kempf, a CRAB* initiative

**Affiliations:** 1 Department of medical oncology, Henri Mondor and Albert Chenevier Teaching Hospital, Assistance Publique – Hôpitaux de Paris, 1 rue Gustave Eiffel, 94000 Créteil, France; 2 Laboratoire Genie Industriel, CentraleSupélec, Paris Saclay University, Gif-sur-Yvette, France; 3 Assistance Publique – Hôpitaux de Paris, Innovation and Data, IT Department, Paris, France; 4 Department of medical informatics, Assistance Publique Hôpitaux de Paris, Centre-Université de Paris (APHP-CUP), Université de Paris, F-75015 Paris, France; 5 Sorbonne Université, Inserm, Université Sorbonne Paris Nord, Laboratoire d’Informatique Médicale et d’Ingénierie des Connaissances pour la e-Santé, LIMICS, Paris, France

**Keywords:** COVID-19, Delivery of health care, Early detection of cancer, Health services research, Neoplasm staging, Neoplasm metastasis

## Abstract

**Background:**

The COVID-19 pandemic might have delayed cancer diagnosis and management. The aim of this systematic review was to compare the initial tumor stage of new cancer diagnoses before and after the pandemic.

**Methods:**

We systematically reviewed articles that compared the tumor stage of new solid cancer diagnoses before and after the initial pandemic waves. We conducted a random-effects meta-analysis to compare the rate of metastatic tumors and the distribution of stages at diagnosis. Subgroup analyses were performed by primary tumor site and by country.

**Results:**

From 2,013 studies published between January 2020 and April 2022, we included 58 studies with 109,996 patients. The rate of metastatic tumors was higher after the COVID-19 outbreak than before (pooled OR: 1.29 (95% CI, 1.06-1.57), *I*^*2*^: 89% (95% CI, 86-91)). For specific cancers, common ORs reached statistical significance for breast (OR: 1.51 (95% CI 1.07-2.12)) and gynecologic (OR: 1.51 (95% CI 1.04-2.18)) cancers, but not for other cancer types. According to countries, common OR (95% CI) reached statistical significance only for Italy: 1.55 (1.01-2.39) and Spain:1.14 (1.02-1.29). Rates were comparable for stage I-II versus III-IV in studies for which that information was available, and for stages I-II versus stage III in studies that did not include metastatic patients.

**Conclusions:**

Despite inter-study heterogeneity, our meta-analysis showed a higher rate of metastatic tumors at diagnosis after the pandemic. The burden of social distancing policies might explain those results, as patients may have delayed seeking care.

**Supplementary Information:**

The online version contains supplementary material available at 10.1186/s12885-023-11795-1.

## Background

In 2020, the COVID-19 pandemic disrupted healthcare systems worldwide. In cancer care, screening programs were suspended in many countries, and care strategies were sometimes adapted to avoid exposing patients to COVID-19 infection, and to reduce the burden on intensive care units. Patients may also have avoided consulting for fear of being contaminated. As a result, screening decreased by 40 to 50%, and cancer diagnoses fell by 27% in January-October 2020 compared to the pre-COVID-19 period [1, 2].

Although recovery plans have been implemented in many countries, it is possible that patients with new cancers whose initial care was delayed could present more advanced tumors, with poorer prognosis. Indeed, modeling studies have anticipated thousands of additional cancer-related deaths in the coming years due to delays in diagnosis and treatment, resulting in tens of thousands of total years of life lost compared with pre-pandemic setting [3].

The aim of this systematic review of the literature with meta-analysis was to compare the proportion of metastatic presentations, and the distribution of initial tumor stage at diagnosis, before and after the COVID-19 outbreak, for patients with solid cancers.

## Materials and methods

This systematic review was reported in accordance with the Preferred Reporting Items for Systematic Review and Meta-Analyses (PRISMA 2020) and the Meta-Analysis of Observational Studies in Epidemiology (MOOSE) guidelines ([4, 5]).

### Data sources, literature searches and eligibility criteria

We searched PubMed and Embase databases for English-language original articles published between January 2020 and April 2022 that included information on the impact of the COVID-19 pandemic on solid malignant tumor stage, using Medical Subject Headings (MeSH) terms and free words. The complete search equation is available in Supplementary materials, Appendix S1. We screened the list of retrieved articles by evaluating titles first, then abstracts, and finally full texts. At each step, two independent investigators evaluated each article. A third independent investigator settled disagreements. We included articles in English that compared cancer stages at diagnosis before versus after the Covid-19 outbreak, using any relevant cancer staging guideline. We included only studies of adult patients, with solid malignant tumors. We excluded reviews, editorials, posters, letters, and guidelines. A reminder list of inclusion and exclusion criteria are available in Appendix S2.

### Data collection and risk of bias assessment

For each article, two independent investigators collected items of interest which are summarized in Appendix S3. In the event of a discrepancy, a third independent investigator settled the issue. We did not contact any study author. We classified primary tumor types as displayed in Appendix S4. We assessed the risk of bias of the included studies with the NIH Quality Assessment Tool for Observational Cohort and Cross-sectional Studies (https://www.nhlbi.nih.gov/health-topics/study-quality-assessment-tools).

### Data analysis

We used the rate of metastatic tumors for comparing cancer presentation before and after the initial COVID-19 pandemic waves. We calculated odds ratio (OR) and 95% confidence intervals (CI) for each study, and we then pooled these individual ORs using a random-effects meta-analysis. Subgroup analyses were performed according to the primary tumor site (when this information was available), and to the study country.

We conducted meta-analysis across all studies and at the subgroup level using the Mantel-Haenszel method. Because we expected heterogeneity between studies, we used the Hartung-Knapp method to calculate CIs on the main effect estimate, with a variance correction [6, 7]. We computed prediction intervals for exposure effect based on Hartung and Knapp’s method [8]. Results were graphically represented in forest plots. The extent of interstudy heterogeneity and subgroup differences were assessed with the Cochran *I*^*2*^ statistics and X^2^ tests, respectively. Between-study variance Tau2 was assessed using the Sidik and Jonkman’s approach and the Q-Profile method for Tau2’s CI [9, 10]. We applied a continuity correction of 0.5 in studies with zero events in one arm.

When articles mentioned missing data or unknown status for some patients, we ignored those patients. When studies overlapped, we only included the study with the broadest inclusion criteria [11]. In situ tumors were excluded from the analysis.

For analyses where more than ten studies could be included, we plotted funnel plots and conducted Thompson and Sharp’s arcsine test to assess the presence of small study effects [12, 13].

Using the same methods, we performed meta-analysis on the rate of stage I-II versus III-IV for the studies where this information was available. When studies mentioned ‘advanced stages’ with no more detailed information, we counted the ‘advanced’ patients as stage IV when a separate ‘locally advanced’ category was also available. These data were then included in the first analysis of metastatic vs non-metastatic status. If no separate ‘locally advanced’ category was available alongside ‘advanced’, we could not know if these ‘advanced’ patients were stage III or IV, and these data were included in the ‘stage I-II vs III-IV’ analysis.

Finally, for studies that did not include metastatic patients (e.g., studies that focused on patients undergoing surgery), we performed a separate meta-analysis comparing stage I-II versus stage III.

All analyses were conducted using R v.2.2.2 (The R project for statistical computing, www.r-project.org) and the *meta* package (v6.2.1.) [14]. No ethics committee approval and no patient consent were necessary because the study was restricted to publicly available data.

## Results

### Study characteristics

**Fig. 1:**
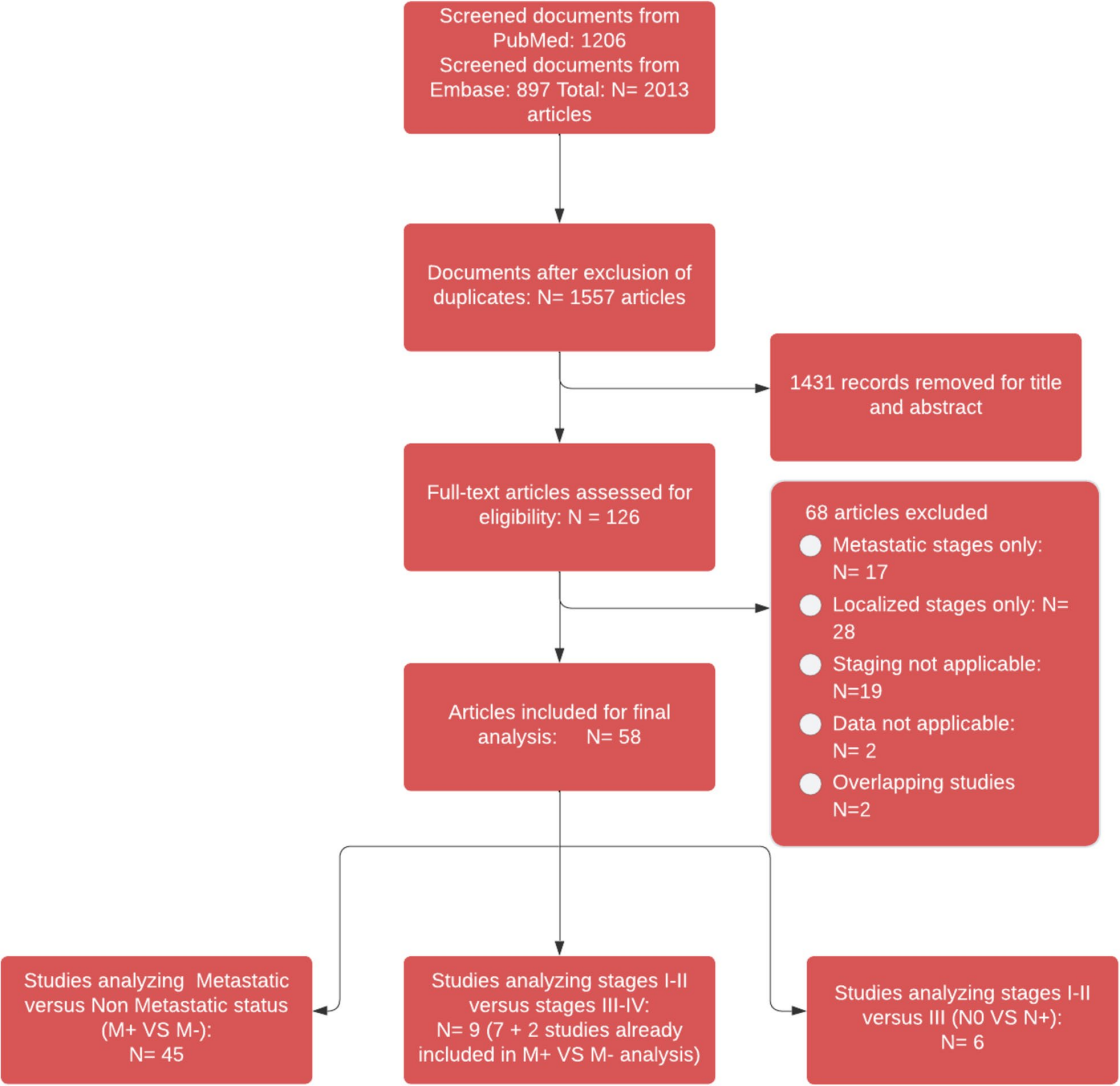
Study selection flowchart

We identified 2,013 studies published between January 2020 and April 2022, and included 58 studies in our meta-analysis ([15–72], Fig. 1, Supplementary Table S1). These articles covered Europe, Asia, North and South America (Supplementary Figure S1). The quality assessment of included studies is presented in Supplementary Table S2. For each study location, lockdowns and cancer screening postponement were summarized in Supplementary Table S3). Breast cancer was the most represented cancer type.

Forty-five studies (98,307 patients) compared metastatic stages IV versus non-metastatic stages I-II-III. Nine studies (7316 patients) compared stages I-II versus III-IV (some articles contributed to both the first and the second analyses) and six (4,373 patients) compared stages I-II versus III without including metastatic patients. The number of patients included per study ranged from 44 to 54,828 (Supplementary Table S1).

Two instances of overlapping studies were identified. In the case of two Dutch register-based studies, we kept the study that included all patients over the one that focused on screening and included only patients aged 50-74 [15, 73]. In the case of two Italian studies, one monocentric and one multicentric that included the previous center, we kept the multicentric study [16, 17].

### Metastatic versus non-metastatic

**Fig. 2:**
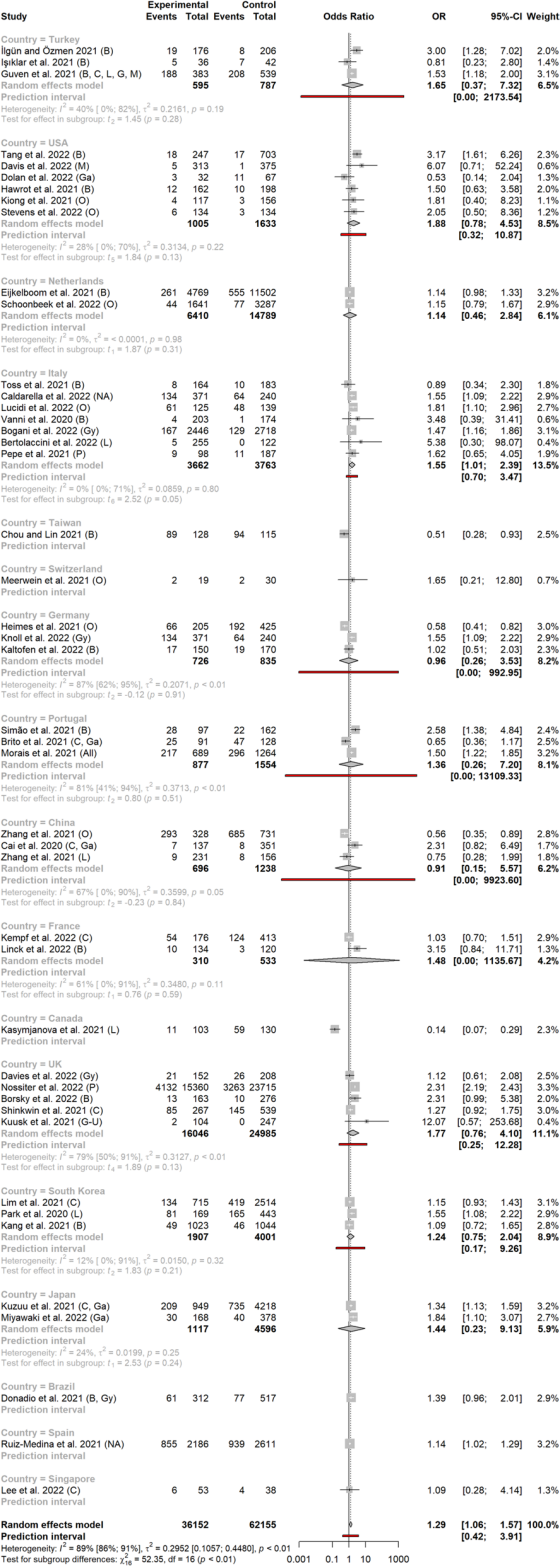
Metastatic tumor rates before and after the Sars Cov2 outbreak, according to study country

**Fig. 3:**
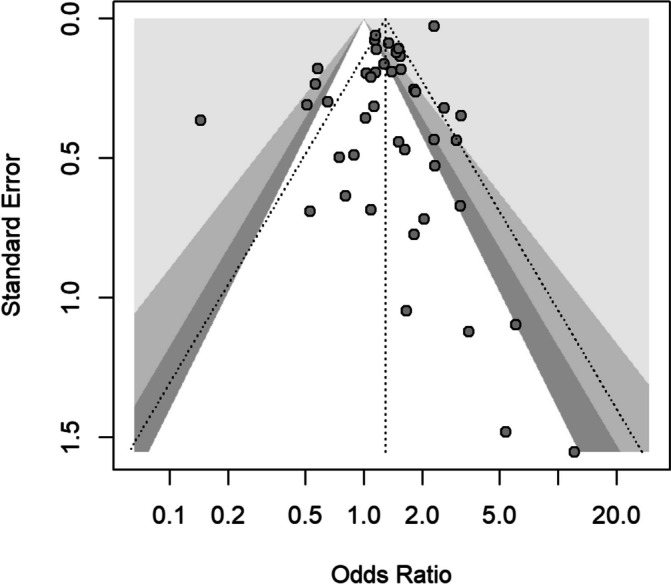
Funnel plot for assessment of study bias

In the 45 studies that contained information on metastatic stage shift, the OR (95% CI) on metastatic stage after vs before the COVID-19 outbreak reached 1.29 (1.06-1.57), indicating a higher probability of patients being metastatic after the outbreak (Fig. 2). Heterogeneity between studies was high, with a *I*^*2*^ of 89% (95% CI, 86-91) and ORs varying from 0.14 to 12.07. Funnel plot showed uneven distribution of small studies (Fig. 3), but the arcsine test was not significant (*p* = 0.25).

In subgroup analysis per country, results reached statistical significance for Italy (seven studies) and Spain (one study), with ORs of 1.55 (1.01-2.39) and 1.14 (1.02-1.29) respectively (Fig. 3). In other countries with more than one study, ORs were 1.65 (0.37-7.32) for Turkey, 1.14 (0.46-2.84) for the Netherlands, 1.88 (0.78-4.53) for the US, 1.48 (0.00-1,135.67) for France, 0.96 (0.26-3.53) for Germany, 1.36 (0.26-7.20) for Portugal, 0.91 (0.15-5.57) for China, 1.77 (0.76-4.10) for the United Kingdom, and 1.24 (0.75-2.04) for South Korea.

**Fig. 4:**
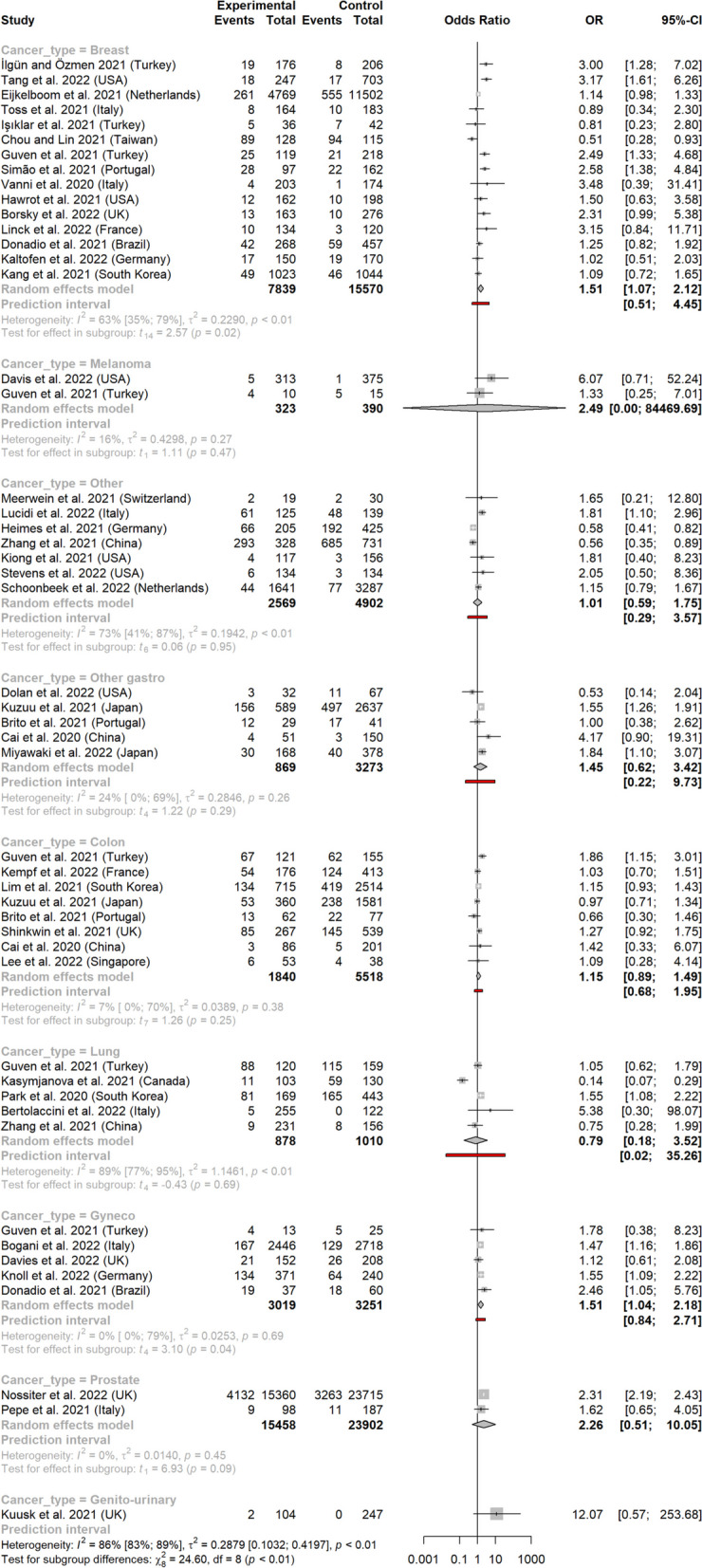
Metastatic tumor rates before and after the pandemic, according to cancer type

In subgroup analysis per location, the related OR reached statistical significance for breast and gynecologic cancers: 1.51 (1.07-2.12) and 1.51 (1.04-2.18) respectively (Fig. 4). ORs for other cancer types did not reach statistical significance: 0.79 (0.18-3.52) for lung cancer, 1.15 (0.89-1.49) for colorectal cancer, 1.45 (0.62-3.42) for other types of digestive cancers, 2.26 (0.51-10.05) for prostate cancer, 12.07 (0.57-253.68) for genito-urinary cancer (one study only), 2.49 (0.00-84,469.69) for melanomas, and 1.01 (0.59-1.75) for other types of cancers (X^2^ = 24.60, *p*<0.01) (Fig. 4a). The funnel plot for breast cancer (the only cancer type with more than ten studies) is available in Supplementary material (Figure S2). Arcsine test was non-significant (*p* = 0.76).

### Localized versus advanced cancer (Stages I-II vs III-IV)

**Fig. 5:**
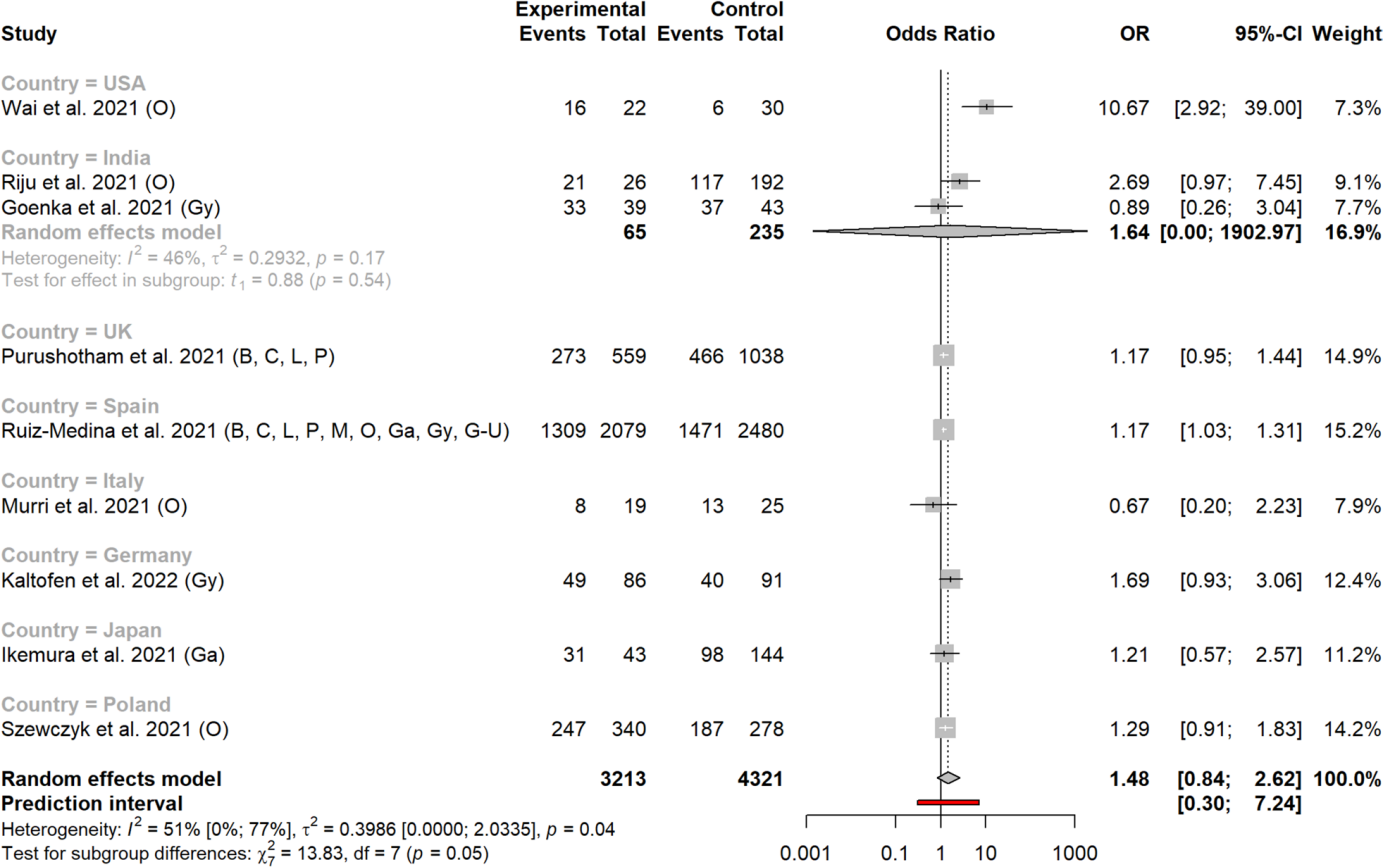
Rates of stage I-II versus III-IV before and after the pandemic according to study country

**Fig. 6:**
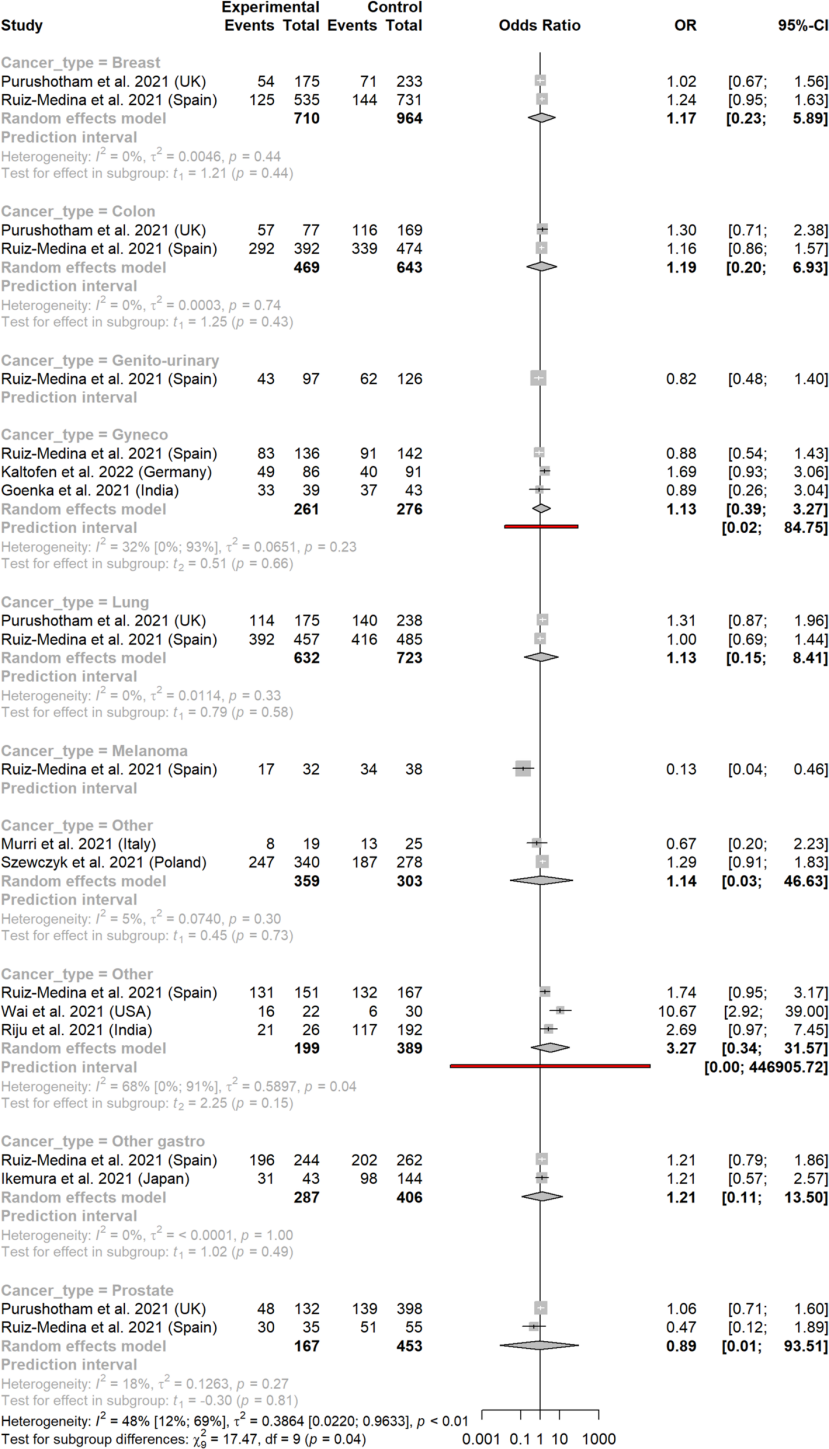
Rates of stage I-II versus III-IV before and after the pandemic according to cancer type

In the analysis of localized versus advanced cancer stages (i.e., stages I-II vs III-IV), the pooled OR was 1.48 (0.84-2.62) (Fig. 5). None of the subgroup analyses per location or per country reached statistical significance (Figs. 5 and 6). Heterogeneity was high, with I^2^ = 51% (0-77) and ORs varying between 0.13 and 10.67

### Studies with no metastatic patients (Stages I-II vs III)

**Fig. 7:**
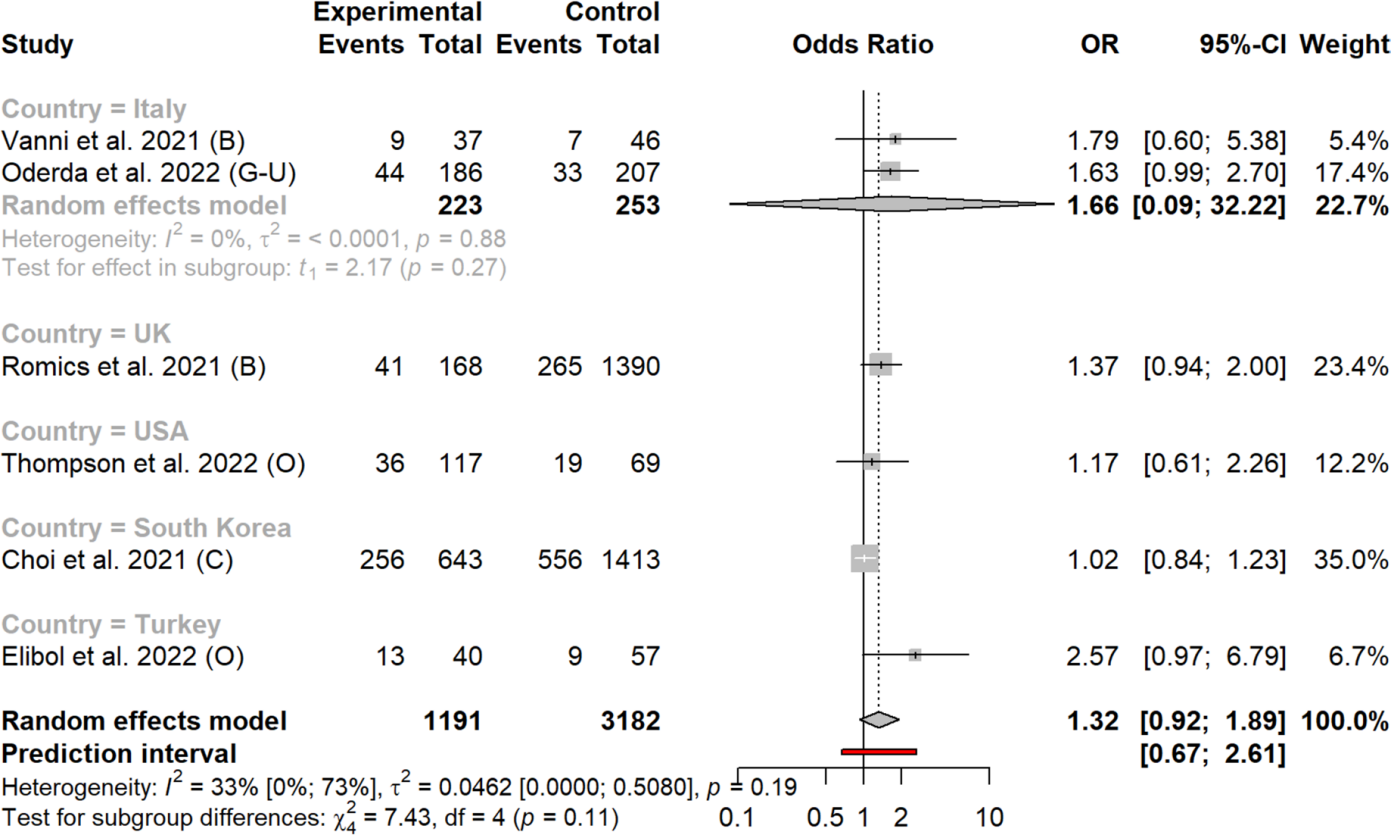
Rates of stage I-II versus III before and after the pandemic according to study country

**Fig. 8:**
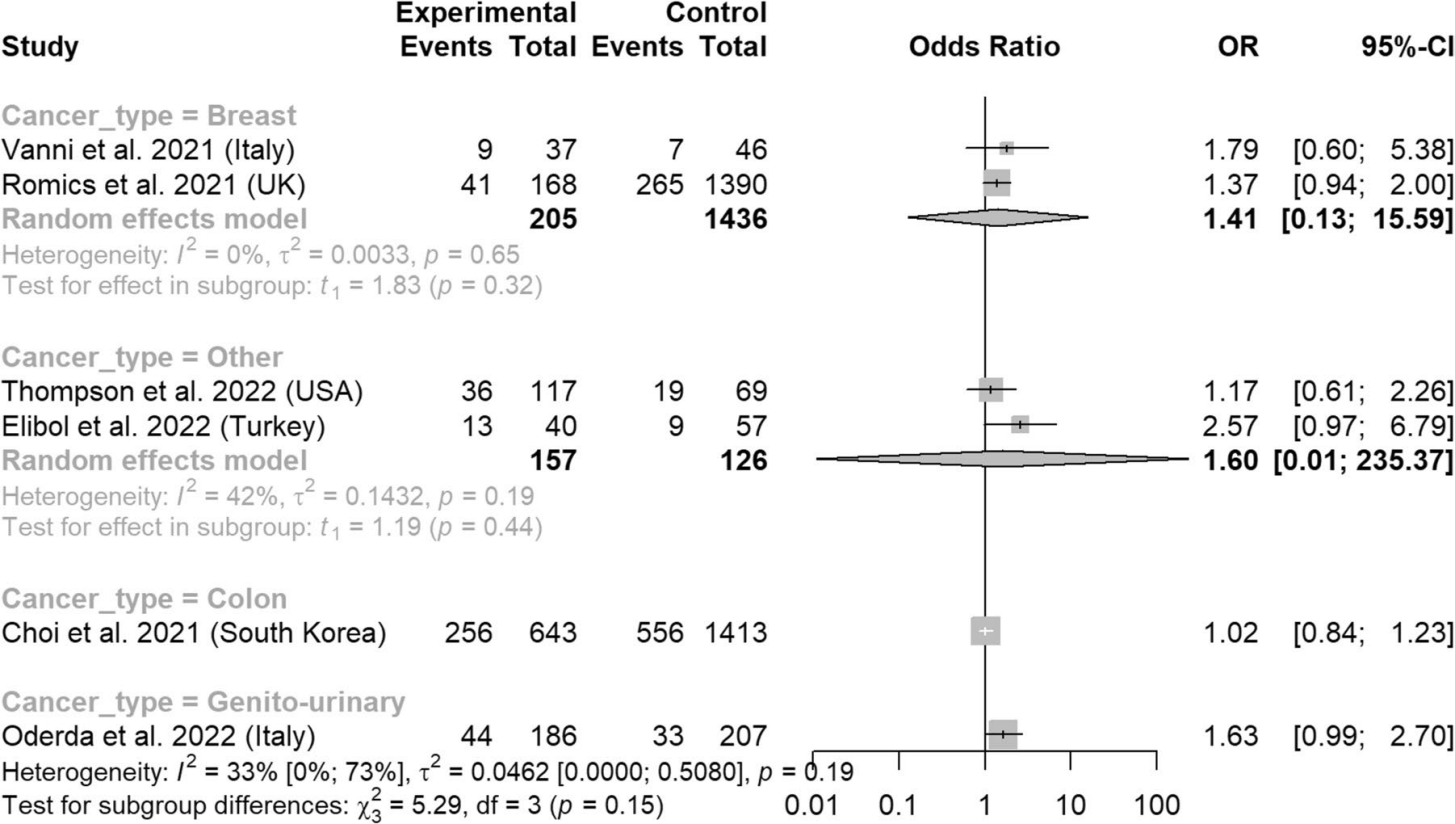
Rates of stage I-II versus III before and after the pandemic, according to cancer type

In the analysis of Stages I-II vs III, in studies that did not include metastatic patients, the pooled OR was 1.32 (0.92-1.89) (Figs. 7 and 8).

## Discussion

We have reviewed published evidence on the impact of the Covid19 outbreak on cancer stages at diagnosis. The results of the main analysis (45 studies) showed an increased rate of metastatic stages at initial clinical presentation, for new solid cancer cases, after compared to before the COVID-19 outbreak. Subgroup analyses yielded significant results for breast and gynecologic cancers, and for Italy and Spain. Secondary analyses on Stages I-II vs III-IV (nine studies) and Stages I-II vs III (for the six studies that excluded metastatic patients) yielded non-significant results.

Based on these results, one may conclude that the pandemic has been associated with more severe forms of cancer at diagnosis. However, we noticed large variations between countries, as well as between tumor locations.

This heterogeneity is still present in more recent observational studies. For example, studies found significant stage shifts for melanomas in the US and Greece, for lung cancer in the UK, for breast cancer in Brazil and for genito-urinary cancers in Iran [74–78]. For colorectal cancer, stage shifts were absent in Canada and the US but noticeable in Italy and South Korea [79–82]. In a systematic review, Pararas et al. analyzed stage shifting for colorectal cancer. They noted a significant increase in the number of patients presenting with de novo metastatic neoplasms during the pandemic (OR 1.65, 95% CI 1.02–2.67) [83]. We found a positive but non-significant association between the Covid19 pandemic and de novo metastatic colorectal cancer, but our analysis included less patients for this location, which may explain the difference in findings.

In our study, Italy and Spain were associated with significant increase of *de novo* metastatic tumor stages. In the case of Italy, this might be due to the number of studies included (seven studies including 7,423 patients, versus five for the UK, six for the US and one to three for other countries). Interruptions to national screening programs could partly explain the excess of metastatic cases observed in Italy [84–86]. We also observed a significant increase in metastatic stages in Spain. However, we included only one Spanish study, limited to Malaga’s region, and more recent Spanish studies have obtained contrasting results [87, 88].

We found a significantly lower presence of metastatic stages at diagnosis after the COVID-19 outbreak in Taiwan and Canada. We included only one small study from each of these countries, so the results should be interpreted with caution. Taiwan drew on its experience of the 2003 SARS pandemic, and applied early policies of travel regulation, testing, and prevention, avoiding lockdowns and screening postponoments [89, 90]. We included a single Canadian study about lung cancer in Quebec. In the same province, Ramanakumar et al. did not find any significant difference in Stage IV for lung cancer before and after the pandemic [91].

We found significantly more metastatic stages at diagnosis after the Covid-19 outbreak for breast and gynecological cancers. In both cases, we included multiple large studies (15 studies with 23,409 patients for breast cancer, 5 studies with 6,270 patients for gynecological cancers). In both cases, interruptions of screening programs may have contributed to the result.

From a general point of view, our results suggest that cancer care disruptions such as national lockdowns and national screening programs postponement led to more severe cancer cases with more metastasis at diagnosis. Unfortunately, these findings only give weight to the dark projections obtained in modelling studies, which anticipate an increase in cancer-related deaths [92–94]. Lockdowns and interruptions to screening programs were probably only one factor contributing to the decrease in diagnoses. Patients feared Covid-19 infection, sometimes more than cancer [95, 96], which can explain the prolonged impact on care seeking behaviors.

We conducted a systematic review of the academic literature, with dual, blinded study selection and data extraction. We covered all cancer types and regions. We also analyzed studies per cancer type and per country, to account for the possibility of different impacts of the pandemic. We included a large number of studies, covering 109,996 patients over 19 countries. However, some limitations must be taken into consideration when considering our findings.

Many studies in our review were small and monocenter. Researchers in areas most affected by the pandemic may have been more prone to report observational data, generating a publication bias (although this was not detected in our analyses). Monocentric studies also cannot account for potential reconfigurations in care trajectories, with some hospitals attracting more cancer patients during the pandemic while others focused on Covid-19 care. Only a minority of studies were population-based, which is the only way to mitigate these issues. When analyzing studies by country, we could not account for variations between regions, including the level of restrictions imposed (e.g., between American states).

We also noticed methodological differences. The way pre- and post-Covid time periods were defined varied between studies. Some studies focused on a short period at the apex of the pandemic, when screening programs stopped, and their area was under lockdown. It is likely that only the most serious patients presented to hospital at these times, increasing the rate of advanced tumors while the absolute number of patients decreased. Other studies defined the COVID-19 period more broadly. Data sources were also heterogeneous in our sample. A few studies were based on registries, which normally guarantee good data completeness and reliability. Other studies mixed data sources, used EHRs or claims data. This may have affected both completeness and quality of the data.

Finally, we only included English sources, and focused on full articles. Data may also have been shared in other languages, and studies presented as conference abstracts may not have been published as full articles.

Despite these limitations, our results suggest that national cancer screening programs should be maintained in high-risk populations even during infectious outbreak waves. After the disruptions, platforms of rapid cancer diagnosis might compensate the interruptions of screening programs and clear diagnosis backlogs. The issue of how cancer care recovers from the pandemic would require population-based studies. Such studies are likely to be available only once registries have been updated, which may take some time [97]. These studies should also look at the evolution of patient survival, given the dark picture painted by modeling studies. Finally, the data we analyzed comes overwhelmingly from high-income countries. Analyzing outcomes in low- and middle-income countries is important to understand how their healthcare systems have worked to mitigate the pandemic impact.

## Conclusion

The COVID-19 outbreak has affected cancer management around the world. This meta-analysis of 58 articles from 19 countries showed an increased rate of metastatic stages at initial clinical presentation for new solid cancer cases diagnosed after the COVID-19 outbreak, with variations between cancer types and between countries. Future studies on the long-term consequences of the pandemic should also assess the impact on patient survival.

### Supplementary Information

**Additional file 1: Figure S1.** Location of included studies. **Figure S2.** Funnel plot for subgroup metastatic vs non-metastatic analysis on breast cancer. **Table S1.** Study characteristics. **Table S2.** Study quality assessment^60^. **Table S3.** Synthesis of first national lockdowns and cancer screening disruptions. **Appendix S1.** Pubmed and EMBASE search equations. **Appendix S2.** Inclusion and exclusion criteria for article selection. **Appendix S3.** List of data elements we extracted from included articles. **Appendix S4.** Classification of primary cancer types.

## Data Availability

The data and the code that support the findings of this study are available on request from the corresponding author.
