# Supplementary material for: Impact of the Sars-Cov-2 outbreak on the initial clinical presentation of new solid cancer diagnoses: a systematic review and meta-analysis
Source: BMC Cancer. 2024 Jan 29;24:143. doi: 10.1186/s12885-023-11795-1 (PMC10823607; doi:10.1186/s12885-023-11795-1)
Supplement: Supplementary file 1 — Additional file 1: Figure S1. Location of included studies. Figure S2. Funnel plot for subgroup metastatic vs non-metastatic analysis on breast cancer. Table S1. Study characteristics. Table S2. Study quality assessment60. Table S3. Synthesis of first national lockdowns and cancer screening disruptions. Appendix S1. Pubmed and EMBASE search equations. Appendix S2. Inclusion and exclusion criteria for article selection. Appendix S3. List of data elements we extracted from included articles. Appendix S4. Classification of primary cancer types. [file 12885_2023_11795_MOESM1_ESM.docx]

Supplementary materials

[Figure S1: Location of included studies 2](#_Toc136438480)

[Figure S2. Funnel plot for subgroup metastatic vs non-metastatic analysis on breast cancer 3](#_Toc136438481)

[Table S1. Study characteristics 4](#_Toc136438482)

[Table S2. Study quality assessment^60^ 20](#_Toc136438483)

[Table S3: Synthesis of first national lockdowns and cancer screening disruptions 23](#_Toc136438484)

[Appendix S1. Pubmed and EMBASE search equations 27](#_Toc136438485)

[Appendix S2. Inclusion and exclusion criteria for article selection 34](#_Toc136438486)

[Appendix S3. List of data elements we extracted from included articles 35](#_Toc136438487)

[Appendix S4. Classification of primary cancer types 36](#_Toc136438488)

## Figure S1: Location of included studies

**
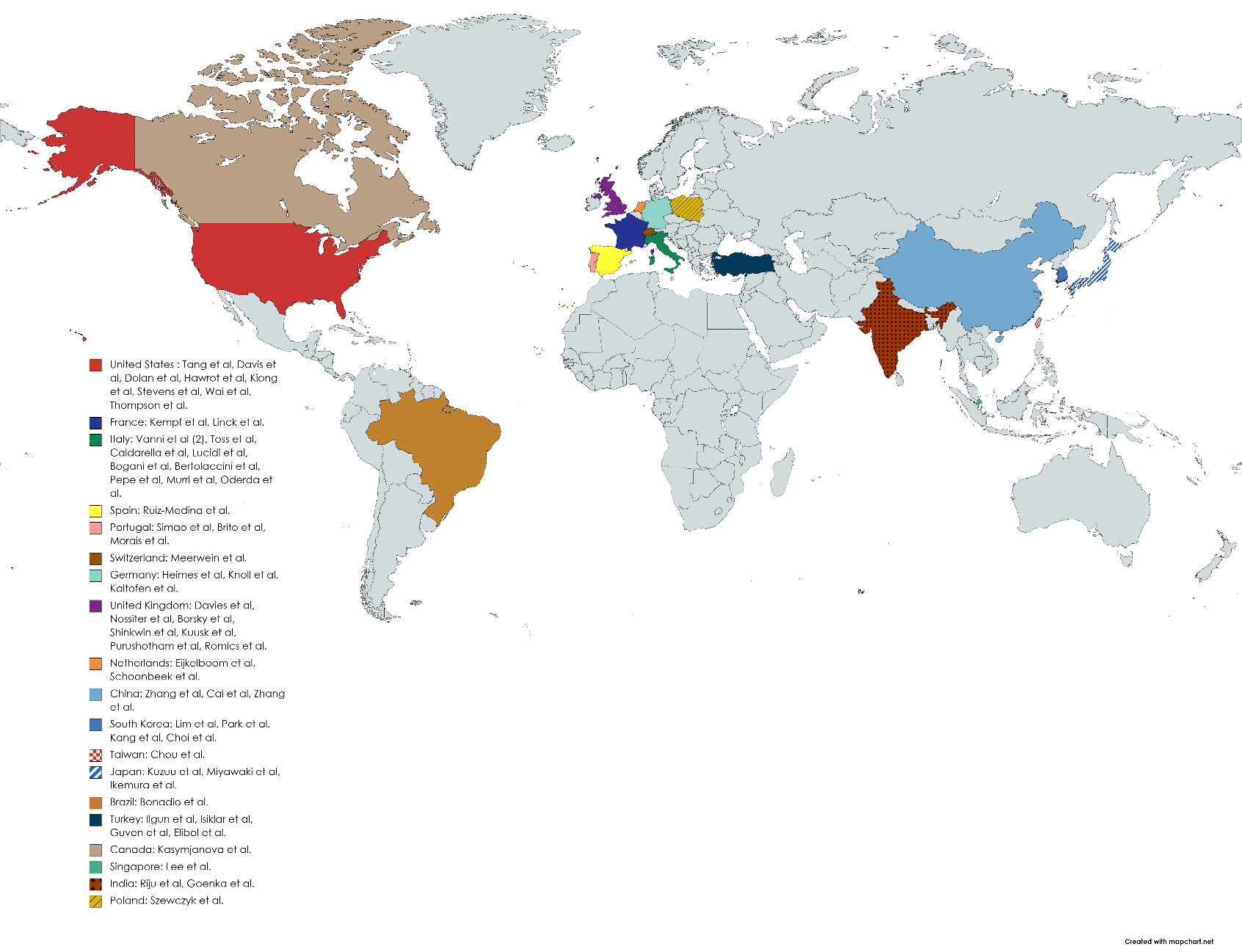
**

## Figure S2. Funnel plot for subgroup metastatic vs non-metastatic analysis on breast cancer


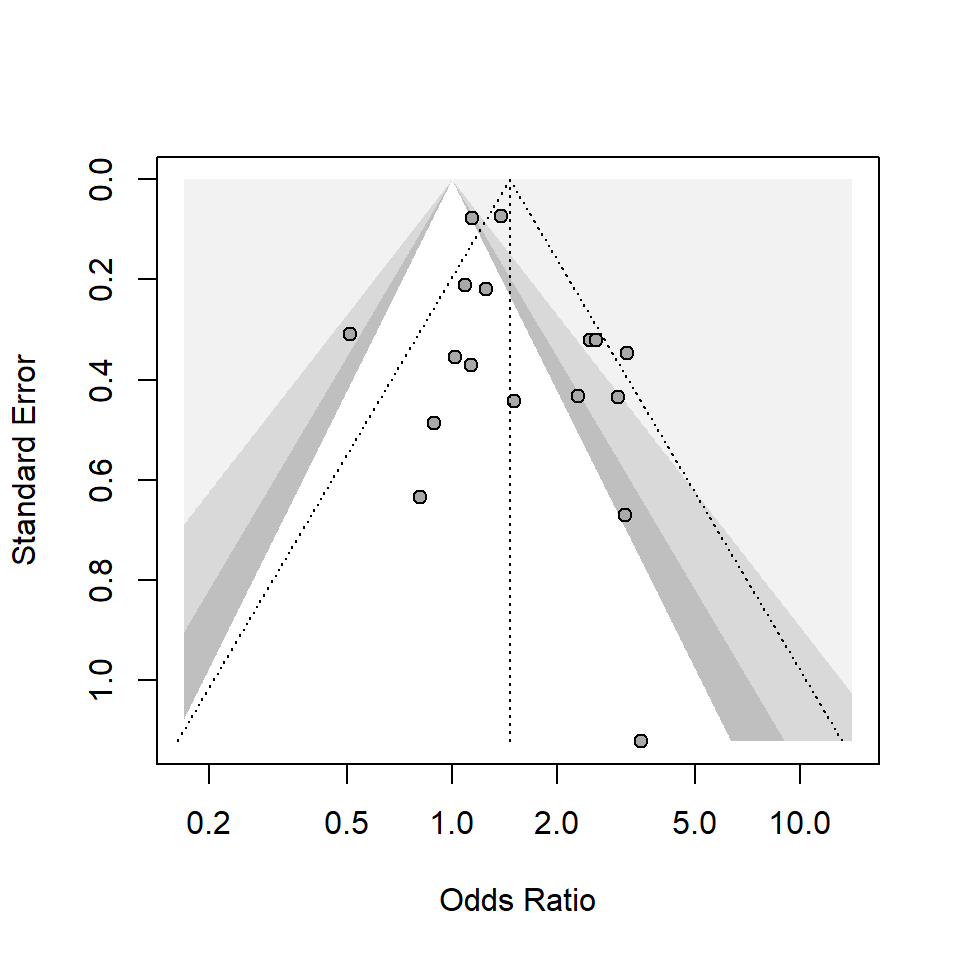


## Table S1. Study characteristics

| **Title** | **Study pop.** | **Control pop.** | **Exposition** | **Staging** |
| --- | --- | --- | --- | --- |
| Changes in gynecologic and breast cancer diagnoses during the first wave of the COVID-19 pandemic: analysis from a tertiary academic gyneco-oncological center in Germany^1^ | patients diagnosed with gynecologic or breast cancer between January and June 2020  N= 236 | Same pop. in 2019  N= 261 | COVID 19 | TNM |
| The impact of COVID-19 pandemic on the rate of newly diagnosed gynecological and breast cancers: a tertiary center perspective^2^ | patients diagnosed with gynecological or breast cancer from March 2020 to April 2020 and November to December 2020  N= 371 | Same periods in 2019  N= 240 | COVID 19 | TNM/ FIGO Stages |
| Has the COVID-19 Pandemic Caused Upshifting in Colorectal Cancer Stage?^3^ | patients diagnosed with colorectal cancer for the first time from January to June 2020.  N= 715 | Same period from 2017 to 2019  N= 2514 | COVID 19 | Stage |
| COVID-19 is Affecting the Presentation and Treatment of Melanoma Patients in the Northeastern United States^4^ | patients undergoing  surgical management for a primary diagnosis of cutaneous  melanoma from May 2020-December 2020  N= 313 | Same pop. from August 2019 to March 2020  N= 375 | COVID 19 | Stage |
| Impact of the COVID-19 pandemic on breast and cervical cancer stage at diagnosis in Brazil^5^ | Patients with histologically confirmed breast or cervical carcinoma of any histologic type.from from 1 September 2020 to 31 January 2021  N= 312 | Same pop. from from 1 September 2019 to 31 January 2020  N= 517 | COVID 19 | Stages |
| The impact of the COVID-19 pandemic on oncological disease extent at FDG PET/CT staging: the ONCOVIPET study^6^ | Only patients≥18 years of age with a cancer diagnosis and FDG-avid tumours, who performed a whole-body  PET/CT for staging purposes for Lung, Gynecologic, Gastro intestinal, lymphoma, breast, head and neck, myeloma, melanoma from June 1 to October 31, 2020.  N=371 | Same pop. from June 1 to October 31, 2019  N= 240 | Covid 19 | TNM |
| Gastrointestinal Cancer Stage at Diagnosis Before and During the COVID-19 Pandemic in Japan^7^ | Patients who were diagnosed with gastrointestinal cancer from March 2020 to December 2020  N=949 | Same pop. from January 2017 to February 2020  N= 4218 | COVID 19 | Stages |
| Significant Decrease in Annual Cancer Diagnoses in Spain during the COVID-19 Pandemic: A Real-Data Study^8^ | All patients diagnosed between  13 March 2020 and 13 March 2021 for 24 cancer types ( cf study )  N= 2340 | Same pop from between 13 March 2019 and 13 March 2020  N= 2825 | COVID 19 | Stages |
| Head and Neck Cancer During Covid-19 Pandemic: Was there a Diagnostic Delay?^9^ | Patients with newly diagnosed laryngeal, salivary, skin of  the head and neck, nasal, paranasal, oral, pharyngeal and  occult cancer from from March 1st, 2020 to October 15th, 2020.  N= 125 | Same pop. from from March 1st, 2019 to October 15th, 2019.  N= 139 | Covid 19 | Stages |
| How Do Breast Cancer Patients Present Following COVID-19 Early Peak in a Breast Cancer Center in Turkey?^10^ | New patients diagnosed for breast cancer between May and June 2020  N= 36 | Same pop. from May and June 2017-2019  N= 42 | COVID 19 | Notion of Early/ Advanced or Metastatic disease. |
| Impact of the COVID-19 pandemic on the diagnosis and treatment of men with prostate cancer^11^ | all patients in England newly diagnosed with prostate cancer between between March, 23 and December, 31 2020  N= 15360 | Same pop. during same period in 2019  N= 23715 | COVID 19 | Stage |
| Study of the gastrointestinal tumor progression during the COVID-19 epidemic in Wuhan^12^ | Patients with gastro intestinal tumor admitted in Wuhan Union Hospital from March To April 2020  N= 137 | Same pop. in same period in 2019.  N= 351 | COVID 19 | pTNM |
| Time to Treatment Initiation for Breast Cancer During the 2020 COVID-19 Pandemic^13^ | New histologic diagnosis of invasive breast cancer, ductal carcinoma in situ  (DCIS), or lobular carcinoma in situ (LCIS) between January  1, 2020, and May 15, 2020  N= 162 | Same pop. between 01/01/2018 and 15/05/2018  N= 198 | COVID 19 | Stage |
| Advanced Stages and Increased Need for Adjuvant Treatments in Breast Cancer Patients: The Effect of the One-year COVID-19 Pandemic^14^ | All patients with a diagnosis of breast cancer from February, 21, 2020 to February, 21 2021  N= 203 | Same pop. from February, 21, 2019, to February, 20, 2020  N= 174 | COVID 19 | TNM |
| Impact of the first surge of the COVID-19 pandemic on a tertiary referral centre for kidney cancer^15^ | Patients underwent renal surgery, from 18 March 2020 until 30 September 2020.  N= 104 | Same pop. from April 2019 to March 2020  N= 247 | COVID 19 | cTNM/ pTNM |
| Prostate Cancer Diagnosis and Management During One Year of the COVID-19 Pandemic^16^ | Men with Pca between March 2020 and March 2021  N= 98 | Same population between March 2019 and 2020  N= 187 | COVID 19 | ISUP |
| Digestive Oncology in the COVID-19 Pandemic Era^17^ | patients with  an inaugural digestive cancer diagnosis from March to August 2020  N= 91 | Same pop. same period In 2018  N= 128 | COVID 19 | Stage |
| The impact of national non-pharmaceutical interventions ('lockdowns') on the presentation of cancer patients^18^ | patients newly diagnosed with cancer in the South East London Cancer Alliance from April 2020 to  September 2020. For lung, prostate, colorectal and breast cancer.  N= 559 | Same pop. from March 2020 to April 2020  N= 1038 | COVID 19 | Notions of Early and Late disease |
| Analysis of Early Impact of COVID-19 on Presentation and Management of Oral Cancers - an Experience from a Tertiary Care Hospital in South India^19^ | All patients primarily concerned with oral cancers and who  underwent primary surgical management for oral cancers with  a curative intend from April 2020 to June 2020  N= 26 | Same pop. from April 2019 to March 2020  N= 192 | COVID 19 | Stage |
| Consequences of the COVID-19 Pandemic and Governmental Containment Policies on the Detection and Therapy of Oral Malignant Lesions-A Retrospective, Multicenter Cohort Study from Germany^20^ | Patients with a histopathological confirmed diagnosis of oral cancer from March, 13, 2020- November 1 2020.  N= 205 | Same pop. in same periods in 2018 and 2019.  N= 425 | COVID 19 | TNM |
| The impact of the COVID-19 pandemic on the short-term survival of patients with cancer in Northern Portugal^21^ | Cases of invasive tumors of the esophagus, skin melanoma, pancreas and prostate diagnosed between 2 March and 1 July 2020  N= 689 | Same pop. in same period in 2019.  N= 1264 | COVID 19 | Stage |
| Delay in Breast Cancer Treatments During the First COVID-19 Lockdown. A Multicentric Analysis of 432 Patients^22^ | Patients with breast surgery from March 11, 2020 to May 30, 2020.  N= 37 | Same pop. in same period from March 11, 2019 to May  30, 2019  N= 46 | COVID 19 | TNM/ Grade |
| Impact of COVID-19 on presentation, staging, and treatment of head and neck mucosal squamous cell carcinoma^23^ | new patients who presented to the head and neck surgical  oncology clinic of Vanderbilt in Nashville from March to July 2020  N= 134 | Same pop. in same period in 2019.  N= 134 | COVID 19 | cTNM/ pTNM |
| The Impact of COVID-19 on the Diagnosis and Treatment of Lung Cancer at a Canadian Academic Center: A Retrospective Chart Review^24^ | patient diagnosed with lung cancer  between 1 March 2020 and 28 February 2021  N= 103 | Same pop. between 1 March 2019 and 29 February 2020.  N= 130 | COVID 19 | Notion of Early / Locoregional and Advanced disease |
| Reduction in healthcare services during the COVID-19 pandemic: Patient screening based on symptoms is an effective strategy for avoiding delayed laryngeal cancer diagnosis^25^ | new histological diagnosis of laryngeal SCC from March 2020 to December 2020.  N= 19 | Same pop. from March 2019 to December 2019.  N= 25 | COVID 19 | Stage |
| The "collateral damage" of the war on COVID-19: impact of the pandemic on the care of epithelial ovarian cancer^26^ | Patient with EOC diagnosed and treated between 1st January 2020 and 31st December 2020.  N= 39 | Same pop. between 1st January 2019 and 30th September 2019.  N= 43 | COVID 19 | Stage |
| Newly diagnosed cancer and the COVID-19 pandemic: tumour stage migration and higher early mortality^27^ | newly referred patients with cancer from March to December 2020. Cancer: breast, CRC, NSCLC, melanoma, cervix.  N= 383 | Same pop. in same period in 2019.  N= 539 | COVID 19 | Stage |
| Lung cancer stage distribution from before COVID-19 through 18 months of the pandemic: the experience of a large-volume oncological referral centre ^28^ | Patients with lung cancer analyzed by pathological staging review by a multidisciplinary  tumour board from January to July 2020 and 2021  N= 255 | Same pop. from January to July 2019.  N= 122 | COVID 19 | Stage |
| COVID-19 pandemic impact on uro-oncological disease outcomes at an Italian tertiary referral center^29^ | Patients treated for suspected or confirmed urologic cancer between January and December 2020.  N= 186 | Same pop. between January and December 2019.  N= 207 | COVID 19 | cTNM |
| Impact of the Coronavirus Disease-2019 Pandemic on Pancreaticobiliary Disease Detection and Treatment^30^ | Newly diagnosed pancreaticobiliary cancer from 7 April to 25 May 2020 AND 26 May to 31 July.  N= 43 | Same pop. from 1 April 2018 to 6 April 2020.  N= 144 | COVID 19 | Stages |
| Impact of Coronavirus Disease 2019 on Clinical Characteristics in Patients With Lung Cancer: A Large Single-Centre Retrospective Study^31^ | patients with lung cancer in Mianyang Central Hospital from February to July 2020.  N= 231 | Same pop. from February to July 2019.  N= 156 | COVID 19 | Stage |
| Thyroid surgery during coronavirus-19 pandemic phases I, II and III: lessons learned in China, South Korea, Iran and Italy^32^ | Patients treated at the Division of Thyroid  Surgery of the China-Japan Union Hospital of Jilin University from January 25 to April 20, 2020  N= 328 | Same pop. from January 25 to April 20, 2019.  N= 731 | COVID 19 | TNM |
| Pattern of breast cancer presentation during the COVID-19 pandemic: results from a cohort study in the UK^33^ | all new breast cancer diagnoses from May to October 2020.  N= 163 | Same pop. from May to October 2019.  N= 276 | COVID 19 | Stage |
| Head and neck surgery during the coronavirus-19 pandemic: The University of California San Francisco experience^34^ | patients undergoing head and neck or reconstructive surgery between March 16, 2020 and April 16, 2020.  N= 22 | Same pop. between March 16, 2019 and April 16, 2019.  N= 30 | COVID 19 | Stage |
| Delayed Breast Cancer Detection in an Asian Country (Taiwan) with Low COVID-19 Incidence^35^ | All patients receiving a breast biopsy between January 21 and July 31, 2020.  N= 115 | Same pop Between January 21 and July 31, 2019.  N= 128 | COVID 19 | Invasive VS Non Invasive |
| Two-month stop in mammographic screening significantly impacts on breast cancer stage at diagnosis and upfront treatment in the COVID era^36^ | women diagnosed with BC in the  province of Modena between May 2020 and July 2020.  N= 164 | Same pop. between May and June 2019.  N= 183 | COVID 19 | Stage |
| Diagnostic pathway and stage migration of sinonasal malignancies in the era of the COVID-19 pandemic^37^ | patients treated for sinonasal malignancies between January 2020 and December 2020.  N= 19 | Same pop between January 2018 and December 2019.  N= 30 | COVID 19 | cTNM/ Stage |
| Impact of the COVID-19 pandemic  on diagnosis, stage, and initial treatment  of breast cancer in the Netherlands:  a population-based study^38^ | Women older than 18 years and diagnosed with breast  cancer during weeks 2–17 of 2020.  N= 4769 | Same pop. during Weeks 2-17 of 2018 and 2019.  N= 11502 | COVID 19 | Stage |
| The Impact of the COVID-19 Pandemic on Breast Cancer Patients^39^ | patients with invasive breast cancer treated between March 2020 and March 2021.  N= 176 | Same pop. between between March 2019 and March 2020.  N= 206 | COVID 19 | Stage |
| Impact of the coronavirus disease 2019 pandemic on first-visit patients with oesophageal cancer in the first infection wave in Saitama prefecture near Tokyo: a single-centre retrospective study^40^ | patients with new oesophageal  cancer who first visited our hospital from April 2020 to March 2021.  N= 168 | Same pop. from April 2018 to March 2020.  N= 378 | COVID 19 | cTNM |
| The impact of the COVID-19 pandemic on the management of head and neck cancer patients at a tertiary care institution in Poland^41^ | all head and neck cancer patients who presented to the multidisciplinary tumour board (MTB) during the 12-month period from March 1, 2020 to February 28, 2021.  N= 340 | Same pop. from February 1, 2019 to February 28, 2020.  N= 278 | COVID 19 | Locally Advanced, Regional, Advanced disease. |
| Characteristics and patterns of care of endometrial cancer before and during COVID-19 pandemic^42^ | newly diagnosed  EC treated in Italy from April 1, 2020 to March 31, 2021.  N= 2446 | Same pop. from March 1, 2019 to February 29, 2020.  N= 2718 | COVID 19 | FIGO Stage |
| Impact of the COVID-19 Pandemic on Surgical Treatment Patterns for Colorectal Cancer in a Tertiary Medical Facility in Korea^43^ | Patients with colon and rectal cancer who underwent  surgery at the Asan Medical Center March to September 2020.  N= 643 | Same pop. from same period in 2019.  N= 1413 | COVID 19 | Stage |
| Esophagectomy for Esophageal Cancer Performed During the Early Phase of the COVID-19 Pandemic^44^ | all patients who underwent esophagectomy for esophageal cancer from March 1, 2020 to June 30, 2020.  N= 32 | Same pop. from January 1,  2019, to December 31, 2019.  N= 67 | COVID 19 | Stage |
| Impact of two waves of Sars-Cov2 outbreak on the number, clinical presentation, care  trajectories and survival of patients newly referred for a colorectal cancer:  A French multicentric cohort study from a large group of University hospitals^45^ | new CRC cases between 1 January 2020 and 31 December 2020.  N= 176 | Same pop. in Same period in 2018 and 2019.  N= 413 | COVID 19 | Stage |
| Impact of the COVID-19 Pandemic on the Diagnosis and Surgery of Breast Cancer: A Multi-Institutional Study^46^ | patients diagnosed with breast cancer from 1 February to 31 July 2020.  N= 1023 | Same pop in same period in 2019.  N= 1044 | COVID 19 | Stage |
| Fewer head and neck cancer diagnoses and faster treatment initiation during COVID-19 in 2020: A nationwide population-based analysis^47^ | all patients with a pathologically confirmed first primary head and neck malignancy from 1 June to 31 December 2020.  N= 1641 | Same pop. in same periods in 2018-2019.  N= 3287 | COVID 19 | Stage |
| Cervical cancer and COVID-an assessment of the initial effect of the pandemic and subsequent projection of impact for women in England: A cohort study^48^ | all cervical cancers between May and October 2020.  N= 152 | Same pop. between May and October 2019.  N= 208 | COVID 19 | Stage |
| The effect of COVID-19 pandemic on laryngeal cancer in a tertiary referral center^49^ | patients who operated due to laryngeal  cancer in a tertiary referral hospital’s ENT department, period not clear.  N= 40 | Same pop, period not clear.  N= 57 | COVID 19 | cTNM |
| Care in the time of COVID-19: impact on the diagnosis and treatment of breast cancer in a large, integrated health care system^50^ | patients with breast cancer diagnosed between March 17 and May 17, 2020.  N= 247 | Same pop. between March 17 and May 17, 2019.  N= 703 | COVID 19 | cTNM/ Grade |
| Collateral effects of the coronavirus disease 2019 pandemic on lung cancer diagnosis in Korea^51^ | patients, aged 18  years or older, who were diagnosed with pathological  lung cancer between February and June 2020.  N= 169 | Same pop between February and  June during 2017–2019.  N= 443. | COVID 19 | Stage |
| COVID-19 and the emergency presentation of colorectal cancer^52^ | Patients with diagnosis of Colorectal adenocarcinoma in 2020.  N= 267 | Same pop. in 2018-2019.  N= 539 | COVID 19 | pTNM |
| Impact of the Novel Coronavirus 2019 (COVID-19) Pandemic on Head and Neck Cancer Care^53^ | Patients over 18 years of age who presented for head and neck  oncologic care from March 18, 2020, to May 20, 2020.  N= 117 | Same pop. in same period in 2019.  N= 69 | COVID 19 | cTNM |
| Did the COVID-19 lockdown result in a delay of colorectal cancer presentation and outcomes? A single centre review^54^ | Initial and revised diagnosis between 7/4/20 and 7/10/20.  N= 53 | Same pop. between 6/10/19 and 6/4/20.  N= 38 | COVID 19 | pTNM |
| What Has Changed During the COVID-19 Pandemic? - The Effect on an Academic Breast Department in Portugal^55^ | Breast cancer patients between March 2020 and March 2021.  N= 97 | Same pop. same period one year previously.  N= 162 | COVID 19 | Stage |
| The impact of COVID-19 on head and neck cancer diagnosis and disease extent^56^ | Patients presenting with newly diagnosed or recurrent HNC from May 14, 2020 to June 18, 2020.  N= 117 | Same pop. in same period from from May 16, 2019 to June  20, 2019.  N= 156 | COVID 19 | cTNM |
| Impact of the COVID-19 lockdown in France on the diagnosis and staging of breast cancers in a tertiary cancer centre^57^ | patients who underwent an imaging-guided percutaneous  breast biopsy performed from January to July 2020.  N= 134 | Same period in 2019.  N= 120 | Covid 19 | TNM |
| A prospective cohort study of the safety of breast cancer surgery during COVID-19 pandemic in the West of Scotland^58^ | People with surgical treatment  for invasive or non-invasive breast cancer between 23 March 2020 and 15 May 2020.  N= 168 | Same pop. in 2015.  N= 1390 | COVID 19 | cTNM/ pTNM/ Grade |

**References:**

1. Kaltofen T, Hagemann F, Harbeck N, et al. Changes in gynecologic and breast cancer diagnoses during the first wave of the COVID-19 pandemic: analysis from a tertiary academic gyneco-oncological center in Germany. *Arch Gynecol Obstet*. Published online 2021. doi:10.1007/S00404-021-06211-7

2. Knoll K, Reiser E, Leitner K, et al. The impact of COVID-19 pandemic on the rate of newly diagnosed gynecological and breast cancers: a tertiary center perspective. *Arch Gynecol Obstet*. Published online 2021. doi:10.1007/S00404-021-06259-5

3. Lim JH, Lee WY, Yun SH, et al. Has the COVID-19 pandemic caused upshifting in colorectal cancer stage? *Ann Coloproctol*. 2021;37(4):253-258. doi:10.3393/AC.2021.00269.0038

4. Davis CH, Ho J, Greco SH, et al. COVID-19 is Affecting the Presentation and Treatment of Melanoma Patients in the Northeastern United States. *Ann Surg Oncol*. Published online 2021. doi:10.1245/S10434-021-11086-8

5. Bonadio RC, Messias AP, Moreira OA, et al. Impact of the COVID-19 pandemic on breast and cervical cancer stage at diagnosis in Brazil. *Ecancermedicalscience*. 2021;15. doi:10.3332/ECANCER.2021.1299

6. Caldarella C, Cocciolillo F, Taralli S, et al. The impact of the COVID-19 pandemic on oncological disease extent at FDG PET/CT staging: the ONCOVIPET study. *Eur J Nucl Med Mol Imaging*. Published online 2021. doi:10.1007/S00259-021-05629-0

7. Kuzuu K, Misawa N, Ashikari K, et al. Gastrointestinal Cancer Stage at Diagnosis before and during the COVID-19 Pandemic in Japan. *JAMA Netw Open*. Published online 2021. doi:10.1001/JAMANETWORKOPEN.2021.26334

8. Ruiz-Medina S, Gil S, Jimenez B, et al. Significant decrease in annual cancer diagnoses in spain during the covid-19 pandemic: A real-data study. *Cancers (Basel)*. 2021;13(13). doi:10.3390/CANCERS13133215

9. Lucidi D, Valerini S, Federici G, Miglio M, Cantaffa C, Alicandri-Ciufelli M. Head and Neck Cancer During Covid-19 Pandemic: Was there a Diagnostic Delay? *Indian Journal of Otolaryngology and Head and Neck Surgery*. Published online 2022. doi:10.1007/S12070-021-03050-5

10. Dauti Işıklar A, Deniz C, Soyder A, Güldoğan N, Yılmaz E, Başaran G. How Do Breast Cancer Patients Present Following COVID-19 Early Peak in a Breast Cancer Center in Turkey? *Eur J Breast Health*. 2021;17(3):253-257. doi:10.4274/EJBH.GALENOS.2021.6161

11. Nossiter J, Morris M, Parry MG, et al. Impact of the COVID-19 pandemic on the diagnosis and treatment of men with prostate cancer. *BJU Int*. 2022;130(2):262-270. doi:10.1111/bju.15699

12. Cai M, Wang GG, Wu Y, Wang Z, Wang GG, Tao K. Study of the gastrointestinal tumor progression during the COVID-19 epidemic in Wuhan. *British Journal of Surgery*. 2020;107(11):e502-e503. doi:10.1002/BJS.11965

13. Hawrot K, Shulman LN, Bleiweiss IJ, et al. Time to Treatment Initiation for Breast Cancer During the 2020 COVID-19 Pandemic. *JCO Oncol Pract*. 2021;17(9):534-540. doi:10.1200/op.20.00807

14. Vanni G, Pellicciaro M, Materazzo M, et al. Advanced stages and increased need for adjuvant treatments in breast cancer patients: The effect of the one-year covid-19 pandemic. *Anticancer Res*. 2021;41(5):2689-2696. doi:10.21873/ANTICANRES.15050

15. Kuusk T, Cullen D, Neves JB, et al. Impact of the first surge of the COVID-19 pandemic on a tertiary referral centre for kidney cancer. *BJU Int*. 2021;128(6):752-758. doi:10.1111/bju.15441

16. Pepe P, Pepe L, Pennisi M, Fraggetta F. Prostate cancer diagnosis and management during one year of the COVID-19 pandemic. *Anticancer Res*. 2021;41(6):3127-3130. doi:10.21873/anticanres.15097

17. Brito M, Laranjo A, Sabino J, Oliveira C, Mocanu I, Fonseca J. Digestive Oncology in the COVID-19 Pandemic Era. *GE Port J Gastroenterol*. 2021;579(5):1-8. doi:10.1159/000514784

18. Purushotham A, Roberts G, Haire K, et al. The impact of national non-pharmaceutical interventions ('lockdowns’) on the presentation of cancer patients. *Ecancermedicalscience*. 2021;15. doi:10.3332/ECANCER.2021.1180

19. Riju J, Tirkey AJ, Mathew M, et al. Analysis of Early Impact of COVID-19 on Presentation and Management of Oral Cancers – an Experience from a Tertiary Care Hospital in South India. *Indian J Surg Oncol*. 2021;12:242-249. doi:10.1007/s13193-021-01302-y

20. Heimes D, Müller LK, Schellin A, et al. Consequences of the COVID-19 pandemic and governmental containment policies on the detection and therapy of oral malignant lesions—a retrospective, multicenter cohort study from germany. *Cancers (Basel)*. 2021;13(12). doi:10.3390/cancers13122892

21. Morais S, Antunes L, Rodrigues J, Fontes F, Bento MJ, Lunet N. The impact of the COVID-19 pandemic on the short-term survival of patients with cancer in Northern Portugal. *Int J Cancer*. 2021;149(2):287-296.

22. Vanni G, Tazzioli G, Pellicciaro M, et al. Delay in breast cancer treatments during the first COVID-19 lockdown. a multicentric analysis of 432 patients. *Anticancer Res*. 2020;40(12):7119-7125. doi:10.21873/anticanres.14741

23. Stevens MN, Patro A, Rahman B, et al. Impact of COVID-19 on presentation, staging, and treatment of head and neck mucosal squamous cell carcinoma. *American Journal of Otolaryngology - Head and Neck Medicine and Surgery*. 2022;43(1). doi:10.1016/J.AMJOTO.2021.103263

24. Kasymjanova G, Anwar A, Cohen V, et al. The impact of COVID-19 on the diagnosis and treatment of lung cancer at a canadian academic center: A retrospective chart review. *Current Oncology*. 2021;28(6):4247-4255. doi:10.3390/CURRONCOL28060360

25. Murri D, Botti C, Bassano E, Fornaciari M, Crocetta FM, Ghidini A. Reduction in healthcare services during the COVID-19 pandemic: Patient screening based on symptoms is an effective strategy for avoiding delayed laryngeal cancer diagnosis. *American Journal of Otolaryngology - Head and Neck Medicine and Surgery*. 2021;42(6). doi:10.1016/J.AMJOTO.2021.103162

26. Goenka L, Anandaradje A, Nakka T, et al. The “collateral damage” of the war on COVID-19: impact of the pandemic on the care of epithelial ovarian cancer. *Medical Oncology*. 2021;38(11). doi:10.1007/s12032-021-01588-6

27. Guven DC, Sahin TK, Yildirim HC, et al. Newly diagnosed cancer and the COVID-19 pandemic: tumour stage migration and higher early mortality. *BMJ Support Palliat Care*. Published online October 28, 2021:bmjspcare-2021-003301. doi:10.1136/BMJSPCARE-2021-003301

28. Bertolaccini L, Ciani O, Prisciandaro E, Sedda G, Spaggiari L. Lung cancer stage distribution from before COVID-19 through 18 months of the pandemic: the experience of a large-volume oncological referral centre. *European Journal of Surgical Oncology*. Published online October 2021. doi:10.1016/J.EJSO.2021.09.024

29. Oderda M, Soria F, Rosi F, et al. COVID-19 pandemic impact on uro-oncological disease outcomes at an Italian tertiary referral center. *World J Urol*. Published online 2021. doi:10.1007/S00345-021-03842-Y

30. Ikemura M, Tomishima K, Ushio M, et al. Impact of the coronavirus disease-2019 pandemic on pancreaticobiliary disease detection and treatment. *J Clin Med*. 2021;10(18). doi:10.3390/JCM10184177

31. Zhang Y, Li J, Li ZK, et al. Impact of Coronavirus Disease 2019 on Clinical Characteristics in Patients With Lung Cancer: A Large Single-Centre Retrospective Study. *Front Oncol*. 2021;11. doi:10.3389/FONC.2021.693002

32. Zhang D, Fu Y, Zhou L, et al. Thyroid surgery during coronavirus-19 pandemic phases I, II and III: lessons learned in China, South Korea, Iran and Italy. *J Endocrinol Invest*. 2021;44(5):1065-1073. doi:10.1007/S40618-020-01407-1

33. Borsky K, Shah K, Cunnick G, Tsang-Wright F. Pattern of breast cancer presentation during the COVID-19 pandemic: results from a cohort study in the UK. *Future Oncology*. 2022;18(4):437-443. doi:10.2217/FON-2021-0970

34. Wai KC, Xu MJ, Lee RH, et al. Head and neck surgery during the coronavirus-19 pandemic: The University of California San Francisco experience. *Head Neck*. 2021;43(2):622-629. doi:10.1002/HED.26514

35. Chou CP, Lin HS. Delayed breast cancer detection in an asian country (Taiwan) with low covid-19 incidence. *Cancer Manag Res*. 2021;13:5899-5906. doi:10.2147/CMAR.S314282

36. Toss A, Isca C, Venturelli M, et al. Two-month stop in mammographic screening significantly impacts on breast cancer stage at diagnosis and upfront treatment in the COVID era. *ESMO Open*. 2021;6(2).

37. Meerwein CM, Stadler TM, Balermpas P, Soyka MB, Holzmann D. Diagnostic pathway and stage migration of sinonasal malignancies in the era of the COVID-19 pandemic. *Laryngoscope Investig Otolaryngol*. 2021;6(5):904-910. doi:10.1002/LIO2.640

38. Eijkelboom AH, de Munck L, Vrancken Peeters MJTFD, et al. Impact of the COVID-19 pandemic on diagnosis, stage, and initial treatment of breast cancer in the Netherlands: a population-based study. *J Hematol Oncol*. 2021;14(1).

39. İlgün AS, Özmen V. The Impact of the COVID-19 Pandemic on Breast Cancer Patients. *Eur J Breast Health*. 2022;18(1):85-90. doi:10.4274/EJBH.GALENOS.2021.2021-11-5

40. Miyawaki Y, Sato H, Lee S, et al. Impact of the coronavirus disease 2019 pandemic on first-visit patients with oesophageal cancer in the first infection wave in Saitama prefecture near Tokyo: a single-centre retrospective study. *Jpn J Clin Oncol*. Published online January 26, 2022. doi:10.1093/JJCO/HYAC002

41. Szewczyk M, Pazdrowski J, Golusiński P, Pazdrowski P, Więckowska B, Golusiński W. The impact of the COVID-19 pandemic on the management of head and neck cancer patients at a tertiary care institution in Poland. *Współczesna Onkologia*. 2021;25(4):264-269. doi:10.5114/WO.2021.111310

42. Bogani G, Scambia G, Cimmino C, et al. Characteristics and patterns of care of endometrial cancer before and during COVID-19 pandemic. *J Gynecol Oncol*. 2022;33(1):e10. doi:10.3802/JGO.2022.33.E10

43. Choi JY, Park IJ, Lee HG, et al. Impact of the COVID-19 pandemic on surgical treatment patterns for colorectal cancer in a tertiary medical facility in Korea. *Cancers (Basel)*. 2021;13(9). doi:10.3390/CANCERS13092221

44. Dolan DP, Swanson SJ, Lee DN, et al. Esophagectomy for Esophageal Cancer Performed During the Early Phase of the COVID-19 Pandemic. *Semin Thorac Cardiovasc Surg*. Published online 2021.

45. Kempf E, Priou S, Lamé G, et al. Impact of two waves of Sars-Cov2 outbreak on the number, clinical presentation, care trajectories and survival of patients newly referred for a colorectal cancer: A French multicentric cohort study from a large group of university hospitals. *Int J Cancer*. Published online 2022. doi:10.1002/IJC.33928

46. Kang YJ, Baek JM, Kim YS, et al. Impact of the COVID-19 Pandemic on the Diagnosis and Surgery of Breast Cancer: A Multi-Institutional Study. *J Breast Cancer*. 2021;24(6):491-503. doi:10.4048/JBC.2021.24.E55

47. Schoonbeek RC, de Jel DVC, van Dijk BAC, et al. Fewer head and neck cancer diagnoses and faster treatment initiation during COVID-19 in 2020: A nationwide population-based analysis: Impact of COVID-19 on head and neck cancer. *Radiotherapy and Oncology*. 2022;167:42-48. doi:10.1016/J.RADONC.2021.12.005

48. Davies JM, Spencer A, Macdonald S, et al. Cervical cancer and COVID—an assessment of the initial effect of the pandemic and subsequent projection of impact for women in England: A cohort study. *BJOG*. 2022;129(7):1133-1139. doi:10.1111/1471-0528.17098

49. Elibol E, Koçak Ö, Sancak M, Arslan B, Gül F, Babademez MA. The effect of COVID-19 pandemic on laryngeal cancer in a tertiary referral center. *European Archives of Oto-Rhino-Laryngology*. Published online 2022. doi:10.1007/S00405-022-07268-Z

50. Tang A, Neeman E, Vuong B, et al. Care in the time of COVID-19: impact on the diagnosis and treatment of breast cancer in a large, integrated health care system. *Breast Cancer Res Treat*. Published online 2022. doi:10.1007/S10549-021-06468-1

51. Park JY, Lee YJ, Kim T, et al. Collateral effects of the coronavirus disease 2019 pandemic on lung cancer diagnosis in Korea. *BMC Cancer*. 2020;20(1). doi:10.1186/S12885-020-07544-3

52. Shinkwin M, Silva L, Vogel I, et al. COVID-19 and the emergency presentation of colorectal cancer. *Colorectal Disease*. 2021;23(8):2014-2019.

53. Thompson JA, Lubek JE, Amin N, et al. Impact of the Novel Coronavirus 2019 (COVID-19) Pandemic on Head and Neck Cancer Care. *Otolaryngology - Head and Neck Surgery (United States)*. 2022;166(1):93-100. doi:10.1177/01945998211004544

54. Lee T, Cheng DZ, Foo FJ, et al. Did the COVID-19 lockdown result in a delay of colorectal cancer presentation and outcomes? A single centre review. *Langenbecks Arch Surg*. Published online January 26, 2022. doi:10.1007/S00423-022-02448-1

55. Simão D, Sardinha M, Reis AF, Spencer AS, Luz R, Oliveira S. What Has Changed During the COVID-19 Pandemic? - The Effect on an Academic Breast Department in Portugal. *Eur J Breast Health*. 2022;18(1):74-78. doi:10.4274/EJBH.GALENOS.2021.2021-11-1

56. Kiong KL, Diaz EMEM, Gross ND, Diaz EMEM, Hanna EY. The impact of COVID-19 on head and neck cancer diagnosis and disease extent. *Head Neck*. 2021;43(6):1890-1897.

57. Linck PA, Garnier C, Depetiteville MP, et al. Impact of the COVID-19 lockdown in France on the diagnosis and staging of breast cancers in a tertiary cancer centre. *Eur Radiol*. Published online 2021. doi:10.1007/S00330-021-08264-3

58. Romics L, Doughty J, Stallard S, et al. A prospective cohort study of the safety of breast cancer surgery during COVID-19 pandemic in the West of Scotland. *Breast*. 2021;55:1-6. doi:10.1016/j.breast.2020.11.015

## Table S2. Study quality assessment^60^

|  | **Q1** | **Q2** | **Q3** | **Q4** | **Q5** | **Q6** | **Q7** | **Q8** | **Q9** | **Q10** | **Q11** | **Q12** | **Q13** | **Q14** |
| --- | --- | --- | --- | --- | --- | --- | --- | --- | --- | --- | --- | --- | --- | --- |
| Kaltofen^1^ | N | Y | NR | Y | N | N | UK | NA | Y | NA | Y | N | UK | N |
| Knoll^2^ | Y | Y | NR | Y | N | N | UK | NA | Y | NA | Y | N | UK | N |
| Lim^3^ | Y | Y | NR | Y | N | N | UK | NA | Y | NA | Y | N | UK | N |
| Davis^4^ | Y | Y | NR | Y | N | N | UK | NA | Y | NA | Y | N | UK | N |
| Bonadio^5^ | Y | Y | NR | Y | N | N | UK | NA | Y | NA | Y | N | UK | N |
| Caldarella^6^ | Y | Y | NR | Y | N | N | UK | NA | Y | NA | Y | N | UK | N |
| Kuzuu^7^ | Y | Y | Y | Y | N | N | UK | NA | Y | NA | Y | N | UK | N |
| Ruiz-Medina^8^ | Y | Y | NR | Y | N | N | UK | NA | Y | NA | Y | N | UK | N |
| Lucidi^9^ | Y | Y | NR | Y | N | N | UK | NA | Y | NA | Y | N | UK | N |
| Işıklar^10^ | Y | Y | NR | N | N | N | UK | NA | Y | NA | Y | N | UK | N |
| Nossiter^11^ | Y | Y | Y | Y | N | N | UK | NA | Y | NA | Y | N | UK | N |
| Cai^12^ | Y | Y | Y | Y | N | N | UK | NA | Y | NA | Y | N | UK | N |
| Hawrot^13^ | Y | Y | Y | N | N | N | UK | NA | Y | NA | Y | N | UK | N |
| Vanni^14^ | Y | Y | NR | Y | N | N | UK | NA | Y | NA | Y | N | UK | N |
| Kuusk^15^ | N | N | NR | Y | N | N | UK | NA | Y | NA | Y | N | UK | N |
| Pepe^16^ | N | N | NR | Y | N | N | UK | NA | Y | NA | Y | N | UK | N |
| Brito^17^ | Y | Y | NR | Y | N | N | UK | NA | Y | NA | N | N | UK | N |
| Purushotham^18^ | Y | Y | NR | Y | N | N | UK | NA | Y | NA | Y | N | UK | N |
| Riju^19^ | N | Y | NR | Y | N | N | UK | NA | Y | NA | Y | N | UK | N |
| Heimes^20^ | N | Y | NR | Y | N | N | UK | NA | Y | NA | Y | N | UK | N |
| Morais^21^ | Y | Y | Y | Y | N | N | UK | NA | Y | NA | Y | N | UK | N |
| Vanni^22^ | Y | Y | NR | Y | N | N | UK | NA | Y | NA | N | N | UK | N |
| Stevens^23^ | Y | Y | Y | Y | N | N | UK | NA | Y | NA | N | N | UK | N |
| Kasymjanova^24^ | Y | Y | NR | Y | N | N | UK | NA | Y | NA | Y | N | UK | N |
| Murri^25^ | Y | Y | NR | Y | N | N | UK | NA | Y | NA | Y | N | UK | N |
| Goenka^26^ | N | Y | NR | N | N | N | UK | NA | Y | NA | Y | N | UK | N |
| Guven ^27^ | Y | Y | NR | Y | N | N | UK | NA | Y | NA | Y | N | UK | N |
| Bertolaccini^28^ | Y | N | NR | NR | N | N | UK | NA | Y | NA | Y | N | UK | N |
| Oderda^29^ | Y | Y | NR | Y | N | N | UK | NA | Y | NA | Y | N | UK | N |
| Ikemura^30^ | N | N | NR | NR | N | N | UK | NA | Y | NA | CD | N | UK | N |
| Zhang^31^ | Y | Y | NR | Y | N | N | UK | NA | Y | NA | Y | N | UK | N |
| Zhang^32^ | Y | Y | NR | Y | N | N | UK | NA | Y | NA | Y | N | UK | N |
| Borsky^33^ | Y | Y | NR | Y | N | N | UK | NA | Y | NA | Y | N | UK | N |
| Wai^34^ | Y | Y | NR | Y | N | N | UK | NA | Y | NA | Y | N | UK | N |
| Chou^35^ | Y | Y | NR | Y | N | N | UK | NA | Y | NA | CD | N | UK | N |
| [Toss](https://pubmed.ncbi.nlm.nih.gov/?term=Toss+A&cauthor_id=33582382)^36^ | Y | Y | NR | Y | N | N | UK | NA | Y | NA | Y | N | UK | N |
| Meerwein^37^ | Y | Y | NR | Y | N | N | UK | NA | Y | NA | Y | N | UK | Y |
| Eijkelboom^38^ | Y | Y | Y | Y | N | N | UK | NA | Y | NA | Y | N | UK | N |
| İlgün^39^ | Y | Y | NR | Y | N | N | UK | NA | Y | NA | Y | N | UK | N |
| Miyawaki^40^ | Y | Y | NR | Y | N | N | UK | NA | Y | NA | Y | N | UK | N |
| Szewczyk^41^ | Y | Y | NR | Y | N | N | UK | NA | Y | NA | Y | N | UK | N |
| Bogani^42^ | Y | Y | NR | Y | N | N | UK | NA | Y | NA | Y | N | UK | N |
| Choi^43^ | Y | Y | NR | Y | N | N | UK | NA | Y | NA | Y | N | UK | N |
| Dolan^44^ | Y | Y | NR | Y | N | N | UK | NA | Y | NA | Y | N | UK | N |
| Kempf^45^ | Y | Y | NR | Y | N | N | UK | NA | Y | NA | Y | N | UK | N |
| Kang^46^ | Y | Y | NR | Y | N | N | UK | NA | Y | NA | Y | N | UK | N |
| Schoonbeek^47^ | Y | Y | NR | Y | N | N | UK | NA | Y | NA | Y | N | UK | N |
| Davies^48^ | Y | Y | NR | Y | N | N | UK | NA | Y | NA | Y | N | UK | N |
| Elibol^49^ | Y | Y | NR | Y | N | N | UK | NA | Y | NA | Y | N | UK | N |
| Tang^50^ | Y | Y | NR | Y | N | N | UK | NA | Y | NA | Y | N | UK | N |
| Park^51^ | Y | Y | NR | Y | N | N | UK | NA | Y | NA | Y | N | UK | N |
| Shinkwin^52^ | Y | Y | NR | Y | N | N | UK | NA | Y | NA | Y | N | UK | N |
| Thompson^53^ | Y | Y | NR | Y | N | N | UK | NA | Y | NA | Y | N | UK | N |
| Lee^54^ | Y | Y | NR | Y | N | N | UK | NA | Y | NA | Y | N | UK | N |
| Simão^55^ | Y | Y | NR | Y | N | N | UK | NA | Y | NA | Y | N | UK | N |
| Kiong^56^ | Y | Y | NR | Y | N | N | UK | NA | Y | NA | Y | N | UK | N |
| Linck^57^ | Y | Y | NR | Y | N | N | UK | NA | Y | NA | Y | N | UK | N |
| Romics^58^ | Y | Y | NR | Y | N | N | UK | NA | Y | NA | N | N | UK | N |

**Abbreviations: Y = Yes, N = No, UK = Unknown NA= Not Applicable NR= Not Reported CD= Can not Determined**

**Questions:**

**1.** Was the research question or objective in this paper clearly stated?

**2.** Was the study population clearly specified and defined?

**3.** Was the participation rate of eligible people at least 50%?

**4.** Were all the subjects selected or recruited from the same or similar populations (including the same time period)? Were inclusion and exclusion criteria for being in the study prespecified and applied uniformly to all participants?

**5.** Was a sample size justification, power description, or variance and effect estimates provided?

**6.** For the analyses in this paper, were the exposure(s) of interest measured prior to the outcome(s) being measured?

**7.** Was the timeframe sufficient so that one could reasonably expect to see an association between exposure and outcome if it existed?

**8.** For exposures that can vary in amount or level, did the study examine different levels of the exposure as related to the outcome (e.g., categories of exposure, or exposure measured as continuous variable)?

**9.** Were the exposure measures (independent variables) clearly defined, valid, reliable, and implemented consistently across all study participants?

**10.** Was the exposure(s) assessed more than once over time?

**11.** Were the outcome measures (dependent variables) clearly defined, valid, reliable, and implemented consistently across all study participants?

**12.** Were the outcome assessors blinded to the exposure status of participants?

**13.** Was loss to follow-up after baseline 20% or less?

**14**. Were key potential confounding variables measured and adjusted statistically for their impact on the relationship between exposure(s) and outcome(s)

60. Smith J, Petrovic P, Rose M, et al. Placeholder Text: A Study. *Citation Styles*. 2021;3. doi:10.10/X

## Table S3: Synthesis of first national lockdowns and cancer screening disruptions

| **Country** | **Date of First National Lockdown** | **Cancer Screening Disruptions** |
| --- | --- | --- |
| Italy | 09/03/2020 – 03/05/2020^61^ | From March to May 2020^61^ |
| Netherlands | from 15 March until early June 2020^62^ | From March, 12, 2020 to mid May 2020^63^ |
| Spain | 14/03/2020 - 09/05/2020^64,65^ | From 12/03/2020 to mid May 2020^66,67^ |
| Turkey | 29/04/2021 – 14/05/2021^68^. No national lockdown in 2020. | From March 2020 to June, 1st 2020 ^69^ |
| USA | Main lockdowns:   - California: 19/03/2020 – 15/06/2021^70^ - Connecticut: 23/03/2020 – 22/04/2020^71,72^ - Illinois: 21/03/2020 – 30/05/2020^73^ - Kansas: 24/03/2020 – 19/04/2020^74^ - Massachusetts: 24/03/2020 – 04/05/2020^75^ - Michigan: 24/03/2020 – 13/04/2020^76^ - New York: 22/03/2020 – 13/06/2020^77^ - Oregon: 24/03/2020 – 15/05/2020^78^ - Wisconsin: 24/03/2020 – 13/05/2020^79^ | Mid March to Mid July 2020^80^ |
| Taïwan | No national lockdown^81^ | No cancer screenings suspension^82^ |
| Switzerland | 17/03/2020 – 27/04/2020^83^ | From mid March 2020 to 6 weeks later^84^ |
| Germany | 22/03/2020 – 20/04/2020^85^ | From late March to late April 2020^86^ |
| Portugal | 18/03/2020 – 02/05/2020^87^ | From mid March to May 2020^87^ |
| China | Main provinces^88^:   - Hubei: 23/01/2020 - 25/03/2020 - Zhejiang: 02/02/2020 – 20/02/2020 - Xinjiang: 18/07/2020 – 26/08/2020 - Hebei: 07/01/2021 – 31/01/2021 - Shaanxi: 22/12/2021 – 16/01/2022 - Henan: 10/01/2022 – 03/02/2022 - Guangdong: 14/03/2022 – 21/03/2022 - Shangai: 01/04/2022 – 01/06/2022 | No clear national datas |
| France | 17/03/2020 – 11/05/2020^89^ | From mid March to 15/06/2020^90^ |
| Canada | Main provinces:   - Ontario: 17/03/2020 – 14/05/2020^91^ - British Columbia: 18/03/2020 – 18/05/2020^92^ - Quebec: 18/03/2020 – 05/04/2020^93^ | 23/03/2020 – 26/05/2020^72^ |
| United Kingdom | 23/03/2020 – 10/05/2020^94^ | Between March and April 2020^95^ |
| South Korea | No national lockdown^96^ | No cancer screening suspension^97^ |
| Japan | No national lockdown^98^ | From April 7 to May 25 2020^99^ |
| Brazil | No national lockdown^100,101^ | No population-based cancer screening programs in Brazil^101^ |
| Singapore | 07/04/2020 – 01/06/2020^102,103^ | 07/04/2020 – 01/06/2020^104^ |
| Poland | 13/03/2023-19/04/2020^105,106^ | April-May 2020^107^ |
| India | 25/03/2020-08/06/2020^108,109^ | During 2020 Lockdown^110^ |

**Sources**

61. Battisti F, Falini P, Gorini G, et al. Cancer screening programmes in Italy during the COVID-19 pandemic: an update of a nationwide survey on activity volumes and delayed diagnoses. *Ann Ist Super Sanita*. 2022;58(1):16-24. doi:10.4415/ANN_22_01_03

62. Yerkes MA, André SCH, Besamusca JW, et al. “Intelligent” lockdown, intelligent effects? Results from a survey on gender (in)equality in paid work, the division of childcare and household work, and quality of life among parents in the Netherlands during the Covid-19 lockdown. *PLoS One*. 2020;15(11):e0242249. doi:10.1371/journal.pone.0242249

63. Dinmohamed AG, Cellamare M, Visser O, et al. The impact of the temporary suspension of national cancer screening programmes due to the COVID-19 epidemic on the diagnosis of breast and colorectal cancer in the Netherlands. *J Hematol Oncol*. 2020;13(1):147. doi:10.1186/s13045-020-00984-1

64. https://www.theguardian.com/world/2020/mar/14/spain-government-set-to-order-nationwide-coronavirus-lockdown. *The Guardian*. March 14, 2020.

65. https://www.bloomberg.com/news/articles/2020-04-22/spain-extends-lockdown-as-virus-cases-rise-again-in-europe#xj4y7vzkg. *Bloomberg*. April 22, 2020.

66. Vives N, Binefa G, Vidal C, et al. Short-term impact of the COVID-19 pandemic on a population-based screening program for colorectal cancer in Catalonia (Spain). *Prev Med (Baltim)*. 2022;155. doi:10.1016/j.ypmed.2021.106929

67. Bosch G, Posso M, Louro J, et al. Impact of the COVID-19 pandemic on breast cancer screening indicators in a Spanish population-based program: a cohort study. 2022;11:77434. doi:10.7554/eLife

68. Genç K. COVID-19 in Turkey: a nation on edge. *Lancet*. 2021;397(10287):1794-1796. doi:10.1016/S0140-6736(21)01098-9

69. Yılmaz E, Güldoğan N, Arıbal E. The effect of covid-19 pandemic on breast imaging: A clinical observations. *Diagnostic and Interventional Radiology*. 2020;26(6):603. doi:10.5152/dir.2020.20644

70. https://www.nytimes.com/2021/04/07/us/california-lockdowns.html. *New York Times*. April 7, 2021.

71. https://www.nbcconnecticut.com/news/coronavirus/coronavirus-outbreak-latest-updates/2242154/. *NBC Connecticut*. March 20, 2020.

72. https://en.wikipedia.org/wiki/COVID-19_lockdowns. Wikipedia.

73. https://www.nbcchicago.com/news/coronavirus/pritzker-expected-to-extend-illinois-stay-at-home-order-thursday-sources/2260588/. *NBC Chicago*. April 23, 2020.

74. https://eu.usatoday.com/story/news/nation/2020/03/21/coronavirus-lockdown-orders-shelter-place-stay-home-state-list/2891193001/. *USA Today News*. March 21, 2020.

75. https://www.newsweek.com/which-state-lockdowns-us-have-been-extended-1497162. *Newsweek*. April 9, 2020.

76. https://www.clickondetroit.com/news/local/2020/03/23/watch-live-michigan-gov-whitmer-to-provide-update-on-states-covid-19-response-efforts/. *Click on Detroit*. March 23, 2020.

77. https://www.cbsnews.com/news/new-york-stay-at-home-extended-coronavirus-lockdown/. *CBS News*. May 15, 2020.

78. https://www.oregonlive.com/coronavirus/2020/05/coronavirus-in-oregon-governor-kate-brown-lays-out-the-road-to-reopening-public-life.html. *The Oregonian*. May 2020.

79. https://www.npr.org/sections/coronavirus-live-updates/2020/05/13/855782006/wisconsin-supreme-court-overturns-the-states-stay-at-home-order. *NPR*. May 13, 2020.

80. https://acsjournals.onlinelibrary.wiley.com/doi/full/10.3322/caac.21692. *ACS Journal*. September 2021.

81. https://www.aier.org/article/lockdowns-in-taiwan-myths-versus-reality/. *American Institute of Economic Research*. May 20, 2021.

82. Tsai HY, Chang YL, Shen CT, Chung WS, Tsai HJ, Chen FM. Effects of the COVID-19 pandemic on breast cancer screening in Taiwan. *Breast*. 2020;54:52-55. doi:10.1016/j.breast.2020.08.014

83.https://web.archive.org/web/20200415104635/https:/www.bag.admin.ch/bag/en/home/krankheiten/ausbrueche-epidemien-pandemien/aktuelle-ausbrueche-epidemien/novel-cov/massnahmen-des-bundes.html. *Internet Archive*. April 20, 2020.

84. https://www.heidi.news/sante/quel-impact-covid-19-a-t-il-eu-sur-la-lutte-contre-le-cancer-du-sein-en-suisse. *Heidi News*. October 1, 2020.

85. https://apnews.com/article/coronavirus-pandemic-health-europe-epidemics-berlin-b61de99739774c1f52b4ba6860054d6d. *AP News*. November 25, 2021.

86. https://www.aerztezeitung.de/Politik/Mammographie-Screening-wegen-Corona-Pandemie-ausgesetzt-408072.html. *AerzteZeitung*. March 26, 2020.

87. Morais S, Antunes L, Rodrigues J, Fontes F, Bento MJ, Lunet N. The impact of the COVID-19 pandemic on the short-term survival of patients with cancer in Northern Portugal. *Int J Cancer*. 2021;149(2):287-296. doi:10.1002/ijc.33532

88. https://en.wikipedia.org/wiki/COVID-19_lockdown_in_China.

89. https://www.gouvernement.fr/info-coronavirus/les-actions-du-gouvernement. *Gouvernement Français*. May 31, 2021.

90. <https://www.ligue-cancer.net/sites/default/files/docs/covid_19_et_cancer_-_2eme_vague_-_positionnement.pdf>. *Ligue contre le cancer*. November 20, 2020.

91. https://www.thestar.com/news/canada/2020/07/17/coronavirus-covid-19-updates-toronto-ontario-gta-canada-july-17-2020.html. *Toronto Star*. July 17, 2020.

92. https://www2.gov.bc.ca/gov/content/covid-19/info/response. *Canadian government*. February 17, 2023.

93. Walker MJ, Wang J, Mazuryk J, et al. Delivery of Cancer Care in Ontario, Canada, During the First Year of the COVID-19 Pandemic. *JAMA Netw Open*. 2022;5(4):e228855. doi:10.1001/jamanetworkopen.2022.8855

94. https://www.instituteforgovernment.org.uk/data-visualisation/timeline-coronavirus-lockdowns. *Institute for government of UK*. April 2023.

95. Greenwood E, Swanton C. Consequences of COVID-19 for cancer care — a CRUK perspective. *Nat Rev Clin Oncol*. 2021;18(1):3-4. doi:10.1038/s41571-020-00446-0

96. Dighe A, Cattarino L, Cuomo-Dannenburg G, et al. Response to COVID-19 in South Korea and implications for lifting stringent interventions. *BMC Med*. 2020;18(1). doi:10.1186/s12916-020-01791-8

97. Lee K, Suh M, Jun JK, Choi KS. Impact of the COVID-19 Pandemic on Gastric Cancer Screening in South Korea: Results From the Korean National Cancer Screening Survey (2017–2021). *J Gastric Cancer*. 2022;22(4):264-272. doi:10.5230/jgc.2022.22.e36

98. https://www.rfi.fr/fr/asie-pacifique/20200407-coronavirus-japon-%C3%A9tat-urge. *RFI*. April 7, 2020.

99. Machii R, Takahashi H. Japanese cancer screening programs during the COVID-19 pandemic: Changes in participation between 2017-2020. *Cancer Epidemiol*. 2023;82. doi:10.1016/j.canep.2022.102313

100. https://www.france24.com/en/americas/20210408-brazil-s-bolsonaro-ignores-call-for-new-lockdown-despite-rise-in-covid-19-cases. *France 24*. April 8, 2021.

101. Ribeiro CM, Correa F de M, Migowski A. Short-term effects of the COVID-19 pandemic on cancer screening, diagnosis and treatment procedures in Brazil: a descriptive study, 2019-2020. *Epidemiol Serv Saude*. 2022;31(1):e2021405. doi:10.1590/S1679-49742022000100010

102. https://www.straitstimes.com/singapore/education/schools-to-shift-to-full-home-based-learning-from-april-8#:~:text=Home-based%20learning%20would%20also%20mean%20the%20cancellation%20of,full%20home-based%20learning%20from%20next%20week%3A%20PM%20Lee.April 14, 2020.

103. https://www.straitstimes.com/singapore/politics/pm-lee-hsien-loong-to-address-nation-on-covid-19-situation-at-noon-on-oct-9.October 21, 2021.

104. Chan JJ, Sim Y, Ow SGW, et al. The impact of COVID-19 on and recommendations for breast cancer care: The Singapore experience. *Endocr Relat Cancer*. 2020;27(9):R307-R327. doi:10.1530/ERC-20-0157

105. https://www.thefirstnews.com/article/poland-in-covid-19-lockdown-pm-orders-bars-restaurants-shopping-centres-and-borders-closed---and-cancels-all-flights-11142. *The First News*. March 20, 2020.

106. https://www.dw.com/en/coronavirus-what-are-the-lockdown-measures-across-europe/a-52905137. *DW*. April 15, 2020.

107. Krok D, Telka E, Szcześniak M, Falewicz A. Threat Appraisal, Resilience, and Health Behaviors in Recovered COVID-19 Patients: The Serial Mediation of Coping and Meaning-Making. *Int J Environ Res Public Health*. 2023;20(4). doi:10.3390/ijerph20043649

108. https://www.financialexpress.com/lifestyle/health/lockdown-5-0-guidelines-state-wise-lockdown-extension-5-0-rules-latest-updates/1975135/. *India News*. May 30, 2020.

109. https://www.bbc.com/news/world-asia-india-52086274. *BBC*. March 30, 2020.

110. Pramesh CS, Chinnaswamy G, Sengar M, Ranganathan P, Badwe R. COVID-19 and cancer care in India. *Nat Cancer*. 2021;2(12):1257-1259. doi:10.1038/s43018-021-00290-w

## Appendix S1. Pubmed and EMBASE search equations

Adapted from PubMed’s ‘Cancer filter’, discontinued in 2019 ^[[1]](#footnote-1)^

**#1**

neoplasms OR American Cancer Society OR angiogenesis inducing agents OR antibodies, neoplasm OR antigens, neoplasm OR antineoplastic agents OR antineoplastic protocols OR biomarkers, tumor OR biopsy OR biopsy OR bone marrow purging OR bone marrow transplantation OR cancer care facilities OR cancer vaccines OR carcinogenicity tests OR carcinogens OR chemoembolization, therapeutic OR clonal evolution OR clonal evolution OR colonography, computed tomographic OR colonoscopy OR colposcopy OR combined modality therapy OR cryosurgery OR cytapheresis OR dna, neoplasm OR drug resistance, neoplasm OR drug screening assays, antitumor OR early detection of cancer OR gene expression regulation, neoplastic OR genes, neoplasm OR graft vs tumor effect OR hematopoietic stem cell transplantation OR hematopoietic stem cell mobilization OR immunotherapy, adoptive OR lymph node excision OR tumor-infiltrating OR mammography OR mastectomy OR medical oncology OR metastasectomy OR mohs surgery OR neoplasm grading OR neoplasm proteins OR neoplasm staging OR neoplasm transplantation OR neoplastic processes OR neoplastic stem cells OR oncogene fusion OR oncogenic viruses OR oncology nursing OR oncology service, hospital OR oncolytic viruses OR papanicolaou test OR papillomavirus vaccines OR peripheral blood stem cell transplantation OR polyomavirus OR radiotherapy OR radiotherapy planning, computer assisted OR rna, neoplasm OR second-look surgery OR SEER program OR stem cell transplantation OR transplantation conditioning OR tumor cells, cultured OR tumor escape OR tumor lysis syndrome OR tumor necrosis factors OR receptors, tumor necrosis factor OR tumor necrosis factor receptor-associated peptides and proteins OR ultrasonography, mammary OR AACR OR AJCC OR (ASCO NOT fungi) OR IARC OR "National Cancer Institute (U.S.)" OR UICC OR aCML OR AGCUS OR AILD OR AML OR ANLL OR ASCUS OR ATLL OR BRCA OR BRCA1 OR BRCA2 OR CIN OR CLL OR CMML OR CMPD OR ECCL OR EGIST OR FMTC OR GLNH OR HNPCC OR HNSCC OR HPV OR HSIL OR ICD O OR JCML OR JMML OR LGLL OR MGUS OR MLH1 OR MPD OR MSH2 OR NSCLC OR RAEB OR RCMD OR SCLC OR VOD OR 5q syndrome OR BCR ABL OR c erbB 2 OR c erbB2 OR carney complex OR cone biopsy OR denys drash OR estrogen receptor negative OR estrogen receptor positive OR li fraumeni OR meigs syndrome OR molar pregnancy OR peutz jeghers OR sentinel lymph node OR struma ovarii OR sturge weber OR zollinger ellison OR (aberrant AND crypt AND foci) OR ((anti-n-methyl-d-aspartate OR anti-nmda) AND encephalitis) OR (barrett AND esophagus) OR (gestational AND trophoblastic) OR (microsatellite AND instability) OR (paget AND (breast OR nipple )) OR (polycythemia AND vera) OR (radiation AND therapy) OR (WAGR AND syndrome) OR (pap AND (smear OR smears )) OR cervical smear OR cervical smears OR pap test OR pap tests OR (PSA AND prostate) OR PSA test OR PSA testing OR (prostate AND specific AND antigen ) OR acanthoma OR acanthomas OR acrochordon OR acrochordons OR acrospiroma OR acrospiromas OR adamantinoma OR adamantinomas OR adenoacanthoma OR adenoacanthomas OR adenoameloblastoma OR adenoameloblastomas OR adenocanthoma OR adenocanthomas OR adenocarcinoma OR adenocarcinomas OR adenofibroma OR adenofibromas OR adenolipoma OR adenolipomas OR adenolymphoma OR adenolymphomas OR adenoma OR adenomas OR adenomatosis OR adenomatous OR adenomyoepithelioma OR adenomyoepitheliomas OR adenomyoma OR adenomyomas OR adenosarcoma OR adenosarcomas OR adenosis OR aesthesioneuroblastoma OR aesthesioneuroblastomas OR ameloblastoma OR ameloblastomas OR amyloidoses OR anaplasia OR androblastoma OR androblastomas OR angioblastoma OR angioblastomas OR angioendothelioma OR angioendotheliomas OR angioendotheliomatosis OR angiofibroma OR angiofibromas OR angiofibrosarcoma OR angiogenesis factor OR angiokeratoma OR angiokeratomas OR angioleiomyoma OR angioleiomyomas OR angiolipoma OR angiolipomas OR angioma OR angiomas OR angiomatosis OR angiomyolipoma OR angiomyolipomas OR angiomyoma OR angiomyomas OR angiomyxoma OR angiomyxomas OR angioreticuloma OR angioreticulomas OR angiosarcoma OR angiosarcomas OR anticancer OR anticarcinogenesis OR anticarcinogenic OR antimutagenesis OR antineoplastic OR antioncogene OR antioncogenes OR antitumor OR antitumors OR antitumour OR antitumours OR apudoma OR apudomas OR argentaffinoma OR argentaffinomas OR arrhenoblastoma OR arrhenoblastomas OR astroblastoma OR astroblastomas OR astrocytoma OR astrocytomas OR astroglioma OR astrogliomas OR atypia OR baltoma OR basiloma OR basilomas OR biochemotherapies OR biochemotherapy OR bioradiotherapy OR Birt-Hogg-Dube OR blastoma OR blastomas OR Buschke-Lowenstein OR cachexia OR cancer OR cancerous OR cancers OR carcinogen OR carcinogenesis OR carcinogenic OR carcinogens OR carcinoid OR carcinoma OR carcinomas OR carcinomatosis OR carcinosarcoma OR carcinosarcomas OR cavernoma OR cavernomas OR cementoma OR cementomas OR cerbB2 OR ceruminoma OR ceruminomas OR chemodectoma OR chemodectomas OR chemoimmunoradiotherapy OR chemoimmunotherapies OR chemoimmunotherapy OR chemoprevention OR chemoradiation OR chemoradiotherapies OR chemoradiotherapy OR cherubism OR chloroma OR chloromas OR cholangiocarcinoma OR cholangiocarcinomas OR cholangiohepatoma OR cholangioma OR cholangiomas OR cholangiosarcoma OR cholesteatoma OR cholesteatomas OR chondroblastoma OR chondroblastomas OR chondroma OR chondromas OR chondrosarcoma OR chondrosarcomas OR chordoma OR chordomas OR chorioadenoma OR chorioadenomas OR chorioangioma OR chorioangiomas OR choriocarcinoma OR choriocarcinomas OR chorioepithelioma OR chorioepitheliomas OR chorionepithelioma OR chorionepitheliomas OR choristoma OR choristomas OR chromaffinoma OR chromaffinomas OR cocarcinogenesis OR collagenoma OR collagenomas OR colonoscopies OR coloscopy OR coloscopies OR comedocarcinoma OR comedocarcinomas OR condyloma OR condylomas OR corticotropinoma OR corticotropinomas OR craniopharyngioma OR craniopharyngiomas OR cylindroma OR cylindromas OR cyst OR cysts OR cystadenocarcinoma OR cystadenocarcinomas OR cystadenofibroma OR cystadenofibromas OR cystadenoma OR cystadenomas OR cystoma OR cystomas OR cystosarcoma OR cystosarcomas OR dentinoma OR dentinomas OR dermatofibroma OR dermatofibromas OR dermatofibrosarcoma OR dermatofibrosarcomas OR dermoid OR desmoid OR desmoplastic OR dictyoma OR dysgerminoma OR dysgerminomas OR dyskeratoma OR dyskeratomas OR dysplasia OR dysplastic OR ectomesenchymoma OR ectomesenchymomas OR elastofibroma OR elastofibromas OR enchondroma OR enchondromas OR enchondromatosis OR endothelioma OR endotheliomas OR ependymoblastoma OR ependymoblastomas OR ependymoma OR ependymomas OR epidermoid OR epithelioma OR epitheliomas OR erythroplasia OR esthesioneuroblastoma OR esthesioneuroblastomas OR esthesioneuroepithelioma OR esthesioneuroepitheliomas OR exostosis OR fibroadenoma OR fibroadenomas OR fibroadenosarcoma OR fibroadenosis OR fibrochondrosarcoma OR fibroelastoma OR fibroelastomas OR fibroepithelioma OR fibroepitheliomas OR fibrofolliculoma OR fibrofolliculomas OR fibroid OR fibroids OR fibrolipoma OR fibrolipomas OR fibroliposarcoma OR fibroma OR fibromas OR fibromatosis OR fibromyoma OR fibromyomas OR fibromyxolipoma OR fibromyxoma OR fibromyxomas OR fibroodontoma OR fibroodontomas OR fibrosarcoma OR fibrosarcomas OR fibrothecoma OR fibrothecomas OR fibroxanthoma OR fibroxanthomas OR fibroxanthosarcoma OR fibroxanthosarcomas OR ganglioblastoma OR ganglioblastomas OR gangliocytoma OR gangliocytomas OR ganglioglioma OR gangliogliomas OR ganglioneuroblastoma OR ganglioneuroblastomas OR ganglioneurofibroma OR ganglioneurofibromas OR ganglioneuroma OR ganglioneuromas OR gastrinoma OR gastrinomas OR germinoma OR germinomas OR glioblastoma OR glioblastomas OR gliofibroma OR gliofibromas OR glioma OR gliomas OR gliomatosis OR glioneuroma OR glioneuromas OR gliosarcoma OR gliosarcomas OR glomangioma OR glomangiomas OR glomangiomatosis OR glomangiomyoma OR glomangiomyomas OR glomangiosarcoma OR glomangiosarcomas OR glucagonoma OR glucagonomas OR gonadoblastoma OR gonadoblastomas OR gonocytoma OR gonocytomas OR granuloma OR granulomas OR granulomatosis OR gynaecomastia OR gynandroblastoma OR gynecomastia OR haemangioblastoma OR haemangioblastomas OR haemangioma OR haemangiomas OR haemangiopericytoma OR haemangiopericytomas OR haemangiosarcoma OR haemangiosarcomas OR hamartoma OR hamartomas OR hemangioblastoma OR hemangioblastomas OR hemangioendothelioma OR hemangioendotheliomas OR hemangioendotheliosarcoma OR hemangioendotheliosarcomas OR hemangioma OR hemangiomas OR hemangiomatosis OR hemangiopericytoma OR hemangiopericytomas OR hemangioperithelioma OR hemangiosarcoma OR hemangiosarcomas OR hepatoblastoma OR hepatoblastomas OR hepatocarcinoma OR hepatocarcinomas OR hepatocholangiocarcinoma OR hepatocholangiocarcinomas OR hepatoma OR hepatomas OR hibernoma OR hibernomas OR hidradenoma OR hidradenomas OR hidrocystoma OR hidrocystomas OR histiocytoma OR histiocytomas OR hodgkin OR hodgkins OR hydatidiform OR hydradenoma OR hydradenomas OR hypernephroma OR hypernephromas OR immunochemoradiotherapy OR immunochemotherapies OR immunochemotherapy OR immunocytoma OR immunocytoma OR immunoradiotherapy OR insulinomas OR integrative oncology OR kasabach-merritt OR keratoacanthoma OR keratoacanthomas OR keratosis OR leiomyoblastoma OR leiomyoblastomas OR leiomyofibroma OR leiomyofibromas OR leiomyoma OR leiomyomas OR leiomyomatosis OR leiomyosarcoma OR leiomyosarcomas OR lipoadenoma OR lipoadenomas OR lipoblastoma OR lipoblastomas OR lipoblastomatosis OR lipoma OR lipomas OR lipomatosis OR liposarcoma OR liposarcomas OR luteinoma OR luteoma OR luteomas OR lymphangioendothelioma OR lymphangioendotheliomas OR lymphangioleiomyomatosis OR lymphangioma OR lymphangiomas OR lymphangiomatosis OR lymphangiomyoma OR lymphangiomyomas OR lymphangiomyomatosis OR lymphangiosarcoma OR lymphangiosarcomas OR lymphoepithelioma OR lymphoepitheliomas OR macroprolactinoma OR malignancies OR malignancy OR malignant OR maltoma OR maltomas OR mammogram OR mammograms OR masculinovoblastoma OR mastocytoma OR mastocytomas OR mcf-7 OR medulloblastoma OR medulloblastomas OR medullocytoma OR medullocytomas OR medulloepithelioma OR medulloepitheliomas OR medullomyoblastoma OR medullomyoblastomas OR melanoacanthoma OR melanoacanthomas OR melanoameloblastoma OR melanocytoma OR melanocytomas OR melanoma OR melanomas OR melanomatosis OR meningioblastoma OR meningioma OR meningiomas OR meningiomatosis OR mesenchymoma OR mesenchymomas OR mesonephroma OR mesonephromas OR mesothelioma OR mesotheliomas OR metaplasia OR metastases OR metastasis OR metastatic OR microcarcinoma OR microcarcinomas OR microglioma OR microgliomas OR micrometastases OR micrometastasis OR mucositis OR myelolipoma OR myelolipomas OR myelosuppression OR myoblastoma OR myoblastomas OR myoepithelioma OR myoepitheliomas OR myofibroblastoma OR myofibroblastomas OR myofibroma OR myofibromas OR myofibromatosis OR myofibrosarcoma OR myofibrosarcomas OR myolipoma OR myolipomas OR myoma OR myomas OR myopericytoma OR myosarcoma OR myosarcomas OR myxofibroma OR myxofibromas OR myxolipoma OR myxolipomas OR myxoliposarcoma OR myxoma OR myxomas OR naevus OR neoplasia OR neoplasia OR neoplasm OR neoplasms OR neoplastic OR nephroblastoma OR nephroblastomas OR neurilemmoma OR neurilemmomas OR neurilemmomatosis OR neurilemoma OR neurilemomas OR neurinoma OR neurinomas OR neuroblastoma OR neuroblastomas OR neurocytoma OR neurocytomas OR neuroepithelioma OR neuroepitheliomas OR neurofibroma OR neurofibromas OR neurofibromatosis OR neurofibrosarcoma OR neurofibrosarcomas OR neurolipocytoma OR neuroma OR neuromas OR neuronevus OR neurothekeoma OR neurothekeomas OR nevus OR nonseminoma OR nonseminomas OR nonseminomatous OR odontoameloblastoma OR odontoma OR oligoastrocytoma OR oligoastrocytomas OR oligodendroglioma OR oligodendrogliomas OR oncocytoma OR oncocytomas OR oncogen OR oncogene OR oncogenes OR oncogenesis OR oncogenic OR oncogens OR oncologic OR oncologist OR oncologists OR oncology OR oncoprotein OR oncoproteins OR opsoclonus-myoclonus OR orchioblastoma OR orchioblastomas OR osteoblastoma OR osteoblastomas OR osteochondroma OR osteochondromas OR osteochondrosarcoma OR osteochondrosarcomas OR osteoclastoma OR osteoclastomas OR osteofibrosarcoma OR osteoma OR osteomas OR osteosarcoma OR osteosarcomas OR pancreatoblastoma OR pancreatoblastomas OR papilloma OR papillomas OR papillomata OR papillomatosis OR papillomavirus OR papillomaviruses OR parachordoma OR parachordomas OR paraganglioma OR paragangliomas OR paraneoplastic OR perineurioma OR perineuriomas OR phaeochromocytoma OR phaeochromocytomas OR pheochromoblastoma OR pheochromoblastomas OR pheochromocytoma OR pheochromocytomas OR pilomatricoma OR pilomatricomas OR pilomatrixoma OR pilomatrixomas OR pinealblastoma OR pinealoblastoma OR pinealoblastomas OR pinealoma OR pinealomas OR pineoblastoma OR pineoblastomas OR pineocytoma OR pineocytomas OR plasmacytoma OR plasmacytomas OR pneumoblastoma OR pneumoblastomas OR pneumocytoma OR polyembryoma OR polyembryomas OR polyhistioma OR polyhistiomas OR polyp OR polyposis OR polyps OR porocarcinoma OR porocarcinomas OR poroma OR poromas OR precancer OR precancerous OR premalignant OR preneoplastic OR prolactinoma OR prolactinomas OR protooncogene OR protooncogenes OR pseudotumor OR pseudotumors OR radiochemotherapy OR radioimmunotherapies OR radioimmunotherapy OR reninoma OR reninomas OR reticuloendothelioma OR reticuloendotheliomas OR reticulohistiocytoma OR reticulohistiocytomas OR reticulosis OR retinoblastoma OR retinoblastomas OR rhabdomyoma OR rhabdomyomas OR rhabdomyosarcoma OR rhabdomyosarcomas OR rhabdosarcoma OR rhabdosarcomas OR sarcoma OR sarcomas OR sarcomatosis OR schwannoma OR schwannomas OR schwannomatosis OR seminoma OR seminomas OR seminomatous OR somatostatinoma OR somatostatinomas OR somatotropinoma OR somatotropinomas OR spermatocytoma OR spiradenoma OR spiradenomas OR spongioblastoma OR spongioblastomas OR steatocystoma OR steatocystomas OR subependymoma OR subependymomas OR syringadenoma OR syringadenomas OR syringocystadenoma OR syringocystadenomas OR syringoma OR syringomas OR teratocarcinoma OR teratocarcinomas OR teratoma OR teratomas OR thecoma OR thecomas OR thymolipoma OR thymolipomas OR trichilemmoma OR trichilemmomas OR trichoadenoma OR trichoblastoma OR trichoblastomas OR trichodiscoma OR trichodiscomas OR trichoepithelioma OR trichoepitheliomas OR trichofolliculoma OR trichofolliculomas OR tricholemmoma OR tricholemmomas OR tumor OR tumorgenesis OR tumorgenic OR tumorigenesis OR tumorigenic OR tumorogenesis OR tumorogenic OR tumors OR tumour OR tumours OR vipoma OR vipomas OR xanthoastrocytoma OR xanthoastrocytomas OR xanthofibroma OR xanthofibromas OR xanthogranuloma OR xanthogranulomas OR xanthoma OR xanthomas OR xanthosarcoma OR xanthosarcomas OR Acta Oncol OR Acta Radiol Oncol Radiat Phys Biol OR Acta Radiol Oncol OR Adv Cancer Res OR Adv Immun Cancer Ther OR Ai Zheng OR Am J Cancer OR Am J Clin Oncol OR Am Soc Clin Oncol Educ Book OR Anal Cell Pathol OR Ann Oncol OR Ann Surg Oncol OR Anti cancer Drugs OR Anticancer Agents Med Chem OR Anticancer Drug Des OR Anticancer Res OR Asia Pac J Clin Oncol OR BMC Cancer OR Baillieres Clin Oncol OR Biochim Biophys Acta OR Blood Cancer J OR Br J Cancer Suppl OR Br J Cancer OR Brain Tumor Pathol OR Breast Cancer Res Treat OR Breast Cancer Res OR Breast Cancer OR Breast J OR Bull Assoc Fr Etud Cancer OR Bull Cancer Radiother OR Bull Cancer OR CA Cancer J Clin OR Can J Oncol OR Can Oncol Nurs J OR Cancer Biochem Biophys OR Cancer Biol Ther OR Cancer Biomark OR Cancer Biother Radiopharm OR Cancer Biother OR Cancer Bull OR Cancer Causes Control OR Cancer Cell Int OR Cancer Cell OR Cancer Cells OR Cancer Chemother Biol Response Modif OR Cancer Chemother Pharmacol OR Cancer Chemother Rep 2 OR Cancer Chemother Rep 3 OR Cancer Chemother Rep OR Cancer Clin Trials OR Cancer Commun OR "Cancer Commun (Lond)" OR Cancer Control OR Cancer Cytol OR Cancer Cytopathol OR Cancer Detect Prev Suppl OR Cancer Detect Prev OR Cancer Discov OR Cancer Drug Deliv OR Cancer Epidemiol Biomarkers Prev OR Cancer Epidemiol OR Cancer Gene Ther OR Cancer Genet OR Cancer Genet Cytogenet OR Cancer Genomics Proteomics OR Cancer Imaging OR Cancer Immun OR Cancer Immunol Immunother OR Cancer Immunol Res OR Cancer Inform OR Cancer Invest OR Cancer J Sci Am OR Cancer J OR Cancer Lett OR Cancer Med OR Cancer Metastasis Rev OR Cancer Microenviron OR Cancer Nurs OR Cancer Pract OR Cancer Prev Control OR Cancer Prev Res Phila OR Cancer Radiother OR Cancer Res Treat OR Cancer Res OR Cancer Sci OR Cancer Surv OR Cancer Treat Rep OR Cancer Treat Res OR Cancer Treat Res Commun OR Cancer Treat Rev OR Cancer OR Carcinogenesis OR Cell Growth Differ OR Cell Oncol Dordr OR Chin Clin Oncol OR Chin J Cancer OR Chin J Cancer OR Clin Breast Cancer OR Clin Cancer Res OR Clin Colorectal Cancer OR Clin Exp Metastasis OR Clin J Oncol Nurs OR Clin Lymphoma Myeloma Leuk OR Clin Lymphoma OR Clin Oncol R Coll Radiol OR Clin Oncol OR Clin Transl Oncol OR CNS Oncol OR Contemp Oncol OR Crit Rev Oncog OR Crit Rev Oncol Hematol OR Curr Cancer Drug Targets OR Curr Oncol Rep OR Curr Oncol OR Curr Opin Oncol OR Curr Probl Cancer OR Curr Treat Options Oncol OR Dimens Oncol Nurs OR Drug Resist Updat OR Eksp Onkol OR Endocr Relat Cancer OR Eur J Cancer B Oral Oncol OR Eur J Cancer Care Engl OR Eur J Cancer Clin Oncol OR Eur J Cancer Prev OR Eur J Cancer OR Eur J Gynaecol Oncol OR Eur J Surg Oncol OR Front Radiat Ther Oncol OR Future Oncol OR Gan No Rinsho OR Gan To Kagaku Ryoho OR Gastric Cancer OR Gastrointest Cancer Res OR Genes Chromosomes Cancer OR Gulf J Oncolog OR Gynecol Oncol OR Head Neck Oncol OR Hematol Oncol Clin North Am OR Hematol Oncol Stem Cell Ther OR Hematol Oncol OR Hered Cancer Clin Pract OR Horm Cancer OR IARC Monogr Eval Carcinog Risk Chem Hum Suppl OR IARC Monogr Eval Carcinog Risk Chem Hum OR IARC Monogr Eval Carcinog Risk Chem Man OR IARC Monogr Eval Carcinog Risks Hum Suppl OR IARC Monogr Eval Carcinog Risks Hum OR IARC Sci Publ OR Important Adv Oncol OR Indian J Cancer OR Infect Agent Cancer OR Innov Oncol Nurs OR Int Adv Surg Oncol OR Int J Biol Markers OR Int J Cancer Suppl OR Int J Cancer OR Int J Clin Oncol OR Int J Gastrointest Cancer OR Int J Gynecol Cancer OR Int J Hyperthermia OR Int J Oncol OR Int J Radiat Oncol Biol Phys OR Int J Surg Oncol OR Integr Cancer Ther OR Invasion Metastasis OR Invest New Drugs OR J Adolesc Young Adult Oncol OR J Assoc Pediatr Oncol Nurses OR J Cancer Educ OR J Cancer Epidemiol Prev OR J Cancer Res Clin Oncol OR J Cancer Res OR J Cancer Surviv OR J Chemother OR J Clin Oncol OR J Community Support Oncol OR J Dermatol Surg Oncol OR J Egypt Natl Canc Inst OR J Environ Pathol Toxicol Oncol OR J Exp Clin Cancer Res OR J Exp Ther Oncol OR J Geriatr Oncol OR J Gynecol Oncol OR J Hematol Oncol OR J Immunother Emphasis Tumor Immunol OR J Immunother OR J Mammary Gland Biol Neoplasia OR J Med Imaging Radiat Oncol OR J Natl Cancer Inst Monogr OR J Natl Cancer Inst OR J Natl Compr Canc Netw OR J Neurooncol OR J Oncol Manag OR J Oncol Pract OR J Oncol OR J Pediatr Hematol Oncol OR J Pediatr Oncol Nurs OR J Soc Integr Oncol OR J Support Oncol OR J Surg Oncol Suppl OR J Surg Oncol OR J Thorac Oncol OR Jaarb Kankeronderz Kankerbestrijd Ned OR JAMA Oncol OR JCO Clin Cancer Inform OR Jpn J Cancer Res OR Jpn J Clin Oncol OR Klin Onkol OR Lancet Oncol OR Leuk Lymphoma OR Leuk Res OR Leukemia OR Lung Cancer OR Lutte Cancer OR Magy Onkol OR Med Oncol Tumor Pharmacother OR Med Oncol OR Med Pediatr Oncol Suppl OR Med Pediatr Oncol OR Melanoma Res OR Mol Cancer Res OR Mol Cancer Ther OR Mol Cancer OR Mol Oncol OR Monogr Neoplast Dis Var Sites OR NCI Monogr OR Nat Rev Cancer OR Nat Rev Clin Oncol OR Natl Cancer Inst Monogr OR Natl Cancer Inst Res Rep OR Neoplasia OR Neoplasma OR Neuro oncol OR Nippon Gan Chiryo Gakkai Shi OR Noshuyo Byori OR Nutr Cancer OR ONS Connect OR ONS News OR Oncogene Res OR Oncogene OR Oncol Nurs Forum OR Oncol Rep OR Oncol Res OR Oncol Res Treat OR Oncologist OR Oncology Huntingt OR Oncology OR Oncotarget OR Onkologie OR Open Clin Cancer J OR Oral Oncol OR Papillomavirus Res OR Pathol Oncol Res OR Pediatr Blood Cancer OR Pediatr Hematol Oncol OR Pigment Cell Melanoma Res OR Pract Radiat Oncol OR Princess Takamatsu Symp OR Proc Am Assoc Cancer Res OR Proc Can Cancer Conf OR Proc Natl Cancer Conf OR Prog Clin Cancer OR Prog Exp Tumor Res OR Prog Tumor Res OR Prostate Cancer Prostatic Dis OR Psychooncology OR Radiat Oncol Investig OR Radiat Oncol OR Radiol Oncol OR Radiother Oncol OR Recent Results Cancer Res OR Rep Carcinog Backgr Doc OR Rev Mex Cir Ginecol Cancer OR S Afr Cancer Bull OR Sci Rep Res Inst Tohoku Univ Med OR Sel Cancer Ther OR Semin Cancer Biol OR Semin Oncol Nurs OR Semin Oncol OR Semin Radiat Oncol OR Semin Surg Oncol OR Semin Urol Oncol OR Strahlenther Onkol OR Suppl J Med Oncol Tumor Pharmacother OR Suppl Tumori OR Support Cancer Ther OR Support Care Cancer OR Surg Oncol Clin N Am OR Surg Oncol OR Symp Fundam Cancer Res OR Target Oncol OR Technol Cancer Res Treat OR Thorac Cancer OR Transl Oncol OR Tumor Res OR Tumori OR Tumour Biol OR Urol Oncol OR Vet Comp Oncol OR Vopr Onkol OR World J Surg Oncol OR Z Krebsforsch Klin Onkol Cancer Res Clin Oncol OR Z Krebsforsch OR Zhongguo Fei Ai Za Zhi OR Zhonghua Zhong Liu Za Zhi

**#2**

**Neoplasm Staging[MeSH Terms] OR Neoplasm Metastasis[MeSH Terms] OR (stage[Title/Abstract] OR metasta*[Title/Abstract] OR pTNM[Title/Abstract] OR TNM[Title/Abstract])**

**#3**

**LitCGeneral[sb]**

**#4**

Address OR Autobiography OR Biography OR Clinical Trial, Veterinary OR Dictionary OR Directory OR Duplicate Publication OR Expression of Concern OR Festschrift OR Historical Article OR Interactive Tutorial OR Interview OR Lecture OR Legal Case OR Legislation OR News OR Newspaper Article OR Observational Study, Veterinary OR Patient Education Handout OR Periodical Index OR Personal Narrative OR Portrait OR Preprint OR Published Erratum OR Randomized Controlled Trial, Veterinary OR Retracted Publication OR Retraction of Publication OR Scientific Integrity Review OR Technical Report OR Video-Audio Media OR Webcast

**#5**

**"animals"[mh:noexp]**

**((#1 *AND* #2) *AND* #3) *NOT* #4 *NOT* #5**

## Appendix S2. Inclusion and exclusion criteria for article selection

**Inclusion criteria:**

- Studies published between January 2020 and April 2022 in PubMed or Embase
- English language only
- Patients >18 years old only
- Solid tumors only
- New cancer diagnosis
- Articles comparing stages before and after the outbreak of the Sars-Cov-2 pandemic

Exclusion criteria:

- Abstracts
- Posters
- Letters to the editor
- Guidelines
- Systematic reviews and other types of literature reviews
- Short communications
- Articles unpublished or from grey literature
- Articles with no or insufficient cancer staging information
- Articles focusing only on pre or post Sars-Cov-2 results (e.g., cross-sectional studies of cancer staging during the pandemic)
- Articles that only included metastatic patients

## Appendix S3. List of data elements we extracted from included articles

1. Identification of articles:

- First author
- Titles
- PMID

1. Method-related items

- Country
- Study type
- Type of data collection (Cancer register, care records, other types)
- Mono or multicentric status
- Type of care center (primary, secondary or tertiary )
- Inclusion criteria
- Cancer type
- Period of interest
- Comparative period
- Notion of cancer screening postponement
- Notion of national lockdown
- Primary outcome
- Secondary outcomes

1. Result-related items

- Number of patients included for each period.
- Median or mean age for each period
- % of male patients foe each period
- Time between diagnosis and treatment for each period
- Cancer staging for each period

## Appendix S4. Classification of primary cancer types

- Breast cancer
- Colorectal cancer
- Other gastro: Esophageal, gastric, pancreatic, biliary tract, liver.
- Gynecologic cancer: Cervix, endometre, ovaries, vulvar/vagina.
- Genito-urinary: Kidney, bladder
- Other cancer: Head and neck (sinonasal, oral, thyroid), sarcoma, germ cells, CUP, mesothelioma.
- Melanoma
- Prostate
- Lungs

1. <https://www.nlm.nih.gov/bsd/pubmed_subsets/cancer_strategy.html>, consulted on 24 May 2023 [↑](#footnote-ref-1)
